# The Plasmin-Sensitive Protein Pls in Methicillin-Resistant *Staphylococcus aureus* (MRSA) Is a Glycoprotein

**DOI:** 10.1371/journal.ppat.1006110

**Published:** 2017-01-12

**Authors:** Isabelle Bleiziffer, Julian Eikmeier, Gottfried Pohlentz, Kathryn McAulay, Guoqing Xia, Muzaffar Hussain, Andreas Peschel, Simon Foster, Georg Peters, Christine Heilmann

**Affiliations:** 1 Institute of Medical Microbiology, University of Münster, Münster, Germany; 2 Interdisciplinary Center for Clinical Research (IZKF), University of Münster, Münster, Germany; 3 Institute for Hygiene, University of Münster, Münster, Germany; 4 Department of Molecular Biology and Biotechnology, University of Sheffield, Sheffield, United Kingdom; 5 Division of Infection, Immunity and Respiratory Medicine, Faculty of Biology, Medicine and Health, University of Manchester, Manchester, United Kingdom; 6 Interfaculty Institute of Microbiology and Infection Medicine, University of Tübingen, Tübingen, Germany; 7 German Center for Infection Research (DZIF), partner site Tübingen, University of Tübingen, Tübingen, Germany; 8 Cluster of Excellence EXC 1003, Cells in Motion, University of Münster, Münster, Germany; University of Washington, UNITED STATES

## Abstract

Most bacterial glycoproteins identified to date are virulence factors of pathogenic bacteria, i.e. adhesins and invasins. However, the impact of protein glycosylation on the major human pathogen *Staphylococcus aureus* remains incompletely understood. To study protein glycosylation in staphylococci, we analyzed lysostaphin lysates of methicillin-resistant *Staphylococcus aureus* (MRSA) strains by SDS-PAGE and subsequent periodic acid-Schiff’s staining. We detected four (>300, ∼250, ∼165, and ∼120 kDa) and two (>300 and ∼175 kDa) glycosylated surface proteins with strain COL and strain 1061, respectively. The ∼250, ∼165, and ∼175 kDa proteins were identified as plasmin-sensitive protein (Pls) by mass spectrometry. Previously, Pls has been demonstrated to be a virulence factor in a mouse septic arthritis model. The *pls* gene is encoded by the staphylococcal cassette chromosome (SCC)*mec* type I in MRSA that also encodes the methicillin resistance-conferring *mecA* and further genes. In a search for glycosyltransferases, we identified two open reading frames encoded downstream of *pls* on the SCC*mec* element, which we termed *gtfC* and *gtfD*. Expression and deletion analysis revealed that both *gtfC* and *gtfD* mediate glycosylation of Pls. Additionally, the recently reported glycosyltransferases SdgA and SdgB are involved in Pls glycosylation. Glycosylation occurs at serine residues in the Pls SD-repeat region and modifying carbohydrates are *N*-acetylhexosaminyl residues. Functional characterization revealed that Pls can confer increased biofilm formation, which seems to involve two distinct mechanisms. The first mechanism depends on glycosylation of the SD-repeat region by GtfC/GtfD and probably also involves eDNA, while the second seems to be independent of glycosylation as well as eDNA and may involve the centrally located G5 domains. Other previously known Pls properties are not related to the sugar modifications. In conclusion, Pls is a glycoprotein and Pls glycosyl residues can stimulate biofilm formation. Thus, sugar modifications may represent promising new targets for novel therapeutic or prophylactic measures against life-threatening *S*. *aureus* infections.

## Author Summary

*Staphylococcus aureus* is a serious pathogen that causes life-threatening infections due to its ability to attach to surfaces, form biofilms, and persist inside the host. One of previously identified virulence factors in *S*. *aureus* pathogenesis is the plasmin-sensitive surface protein Pls. We here identified Pls as a posttranslationally modified glycoprotein and characterized the domain within Pls that becomes glycosylated as well as the modifying sugars. Moreover, we found that the glycosyltransferases GtfC and GtfD carry out the glycosylation reactions. In a search for a role for the modifying sugars, we found that Pls can stimulate biofilm formation apparently via two distinct mechanisms, one being dependent on glycosylation by GtfC and GtfD the other being independent of glycosylation as well as eDNA. Moreover, we found that none of the already known Pls functions is mediated by the sugar moieties. Thus, we conclude that GtfC/GtfD-glycosylated Pls may contribute to MRSA pathogenicity via stimulation of biofilm formation and may serve as future target to combat or prevent infections with this serious pathogen.

## Introduction

Although usually being a common inhabitant of the human skin and mucous membranes, *Staphylococcus aureus* is a human pathogen that can cause diseases ranging from mild skin infections to serious and life-threatening infections, such as endocarditis, osteomyelitis, pneumonia, meningitis, and sepsis [1, 2]. Especially due to the increasing use of various medical devices and implants in modern medicine, the number of nosocomial *S*. *aureus* infections is constantly rising [3, 4]. Furthermore in the past three decades, the emergence of antibiotic-resistant staphylococci, such as methicillin-resistant *S*. *aureus* (MRSA) represents an increasing problem in the treatment of *S*. *aureus* infections. Thus, alternative therapeutic or prophylactic measures against *S*. *aureus* infections are urgently required.

Until recently, it has been considered a dogma that bacteria are unable to glycosylate proteins, because they lack the equivalent cellular structures involved in protein glycosylation in eukaryotes. Now, it is widely accepted that bacteria can glycosylate proteins. Most bacterial glycoproteins identified to date are virulence factors of pathogenic bacteria, i.e. adhesins and invasins [5–7]. Bacteria have two basic systems to glycosylate proteins: *N*-linked and *O*-linked glycosylation [8–10]. The sugar transfer is carried out by glycosyltransferases (Gtfs) [10]. The *N*-linked glycosylation pathways have been well characterized in Gram-negative bacteria [5–7, 9].

Known *O*-linked glycoproteins include serine-rich repeat (SRR) surface proteins from Gram-positive cocci, such as the 286-kDa platelet-binding protein GspB from *Streptococcus gordonii* and the homologous 227-kDa serine-rich adhesin for platelets (SraP) from *S*. *aureus* [11–14]. Very recently, the serine-rich *S*. *aureus* clumping factor A (ClfA) has also been identified as a glycoprotein [15, 16].

Generally, adherence of *S*. *aureus* to components of the extracellular matrix or host tissue, i.e. endothelial and epithelial cells or platelets, is a prerequisite for tissue colonization and the initiation of an infection, such as infective endocarditis. *S*. *aureus* harbors an armamentarium of surface (covalently linked to the peptidoglycan) and surface-associated (non-covalently attached to the surface) adhesins that mediate adherence to extracellular matrix or plasma proteins acting as bridging molecules or directly to host cell receptors [17]. SraP and ClfA belong to a family of staphylococcal surface proteins characterized by common features, such as an N-terminal signal peptide, a ligand-binding A region, a repeat region, and a C-terminal cell wall anchor [18]. The C-terminal anchor domain consists of an LPXTG-motif that is involved in covalent linkage of the protein to peptidoglycan, followed by a stretch of hydrophobic amino acids (aa), and a short charged tail [18].

SraP and GspB have very similar features including their large size, an atypically long putative N-terminal signal peptide, two SRR domains, srr1 and srr2, that are separated by a non-repeat region, and the LPXTG cell wall anchor [13, 14]. Furthermore, both genes, *gspB* and *sraP*, are located in operons that additionally encode accessory secretion (Sec) proteins and Gtfs [19, 20]. Within the accessory *sec* system, *gtfA* and *gtfB* are located downstream of the *sraP* structural gene and have been reported to be required for the glycosylation of SraP [20, 21]. The SraP protein domain containing srr1 and the non-repeat region was found to directly bind to platelets and the expression of *sraP* correlates with an increased virulence in a rabbit model of experimental infective endocarditis [14]. SRR glycoproteins have also been associated with increased virulence in animal models of meningitis [22, 23] and blood stream infection [16, 24]. In contrast to SraP, ClfA is not part of an operon that also contains the genes encoding the Gtfs. Instead, ClfA becomes glycosylated by the novel Gtfs SdgA and SdgB, whose genes are located downstream of the tandemly arranged genes encoding the SD-repeat (Sdr) proteins SdrC, SdrD, and SdrE [15, 16].

The potential role of posttranslational protein glycosylation in adherence or in the pathogenesis of staphylococcal infections in general is largely unknown. Therefore, the aim of this study was to identify *S*. *aureus* surface proteins that are posttranslationally modified by carbohydrate moieties, the underlying glycosylation machinery and their potential role in the pathogenesis of staphylococcal infections. We found that the plasmin-sensitive surface protein Pls previously characterized as a virulence determinant in mouse septic arthritis and associated with the staphylococcal cassette chromosome (SCC)*mec* type I [25, 26] is a glycoprotein and identified two open reading frames downstream of the *pls* structural gene that encode novel Gtfs (termed GtfC/GtfD) involved in Pls glycosylation. Functional characterization indicated that Pls carbohydrate moieties can stimulate biofilm formation, while they are not apparently involved in other Pls properties.

## Results

### Identification of glycosylated proteins in *S*. *aureus*

**Fig 1:**
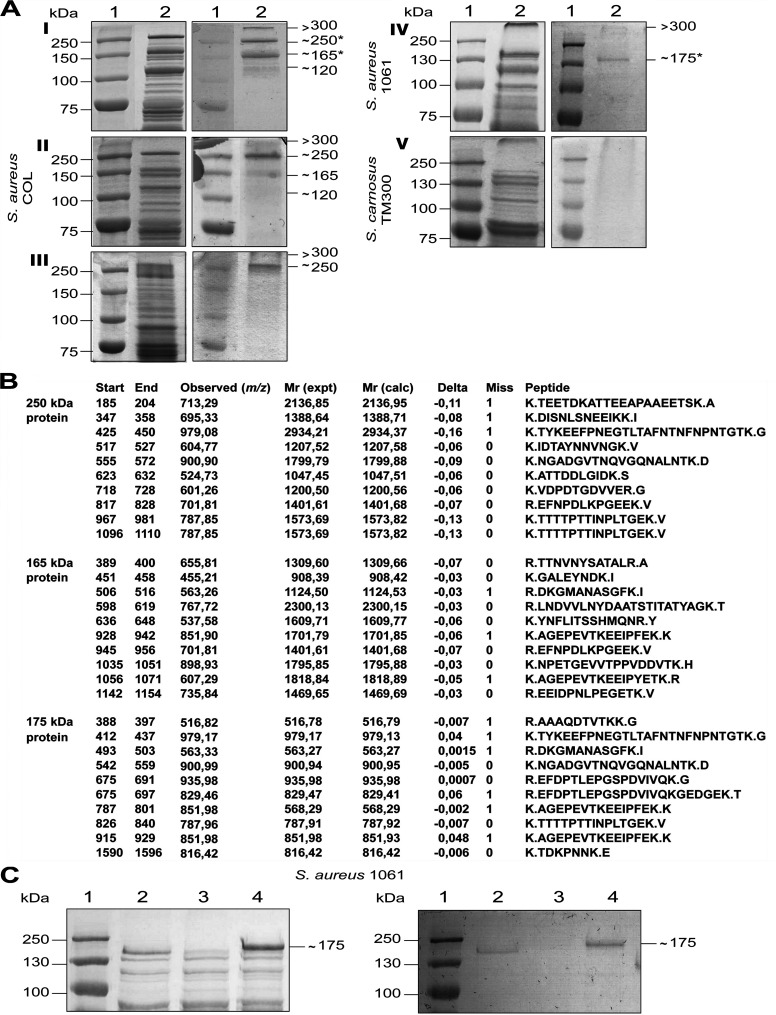
The *S*. *aureus* plasmin-sensitive protein Pls is a glycoprotein. **(A)** For all samples, left panel are Coomassie-Blue stained gels (10% separation gel) corresponding to PAS staining (right panel) to detect glycosylated proteins. Lane 1, marker proteins; Lane 2, surface proteins from I, *S*. *aureus* COL, stationary phase; II, *S*. *aureus* COL, exponential phase, III, *S*. *aureus* COL surface-associated proteins; IV, *S*. *aureus* 1061, stationary phase; V, *S*. *carnosus* TM300 stationary phase. The stars indicate the proteins subjected to MS. **(B)** The 250-kDa, 165-kDa, and 175-kDa proteins were identified as Pls by MS. For each analysis, 10 detected peptides are given with their aa positions (Start, End), observed monoisotopic mass of the respective peptide in the spectrum [Observed (m/z)], experimental mass of the respective peptide calculated from the observed m/z value [Mr (expt)], theoretical mass of the respective peptide based on its sequence [Mr (calc)], difference between the theoretical Mr (calc) and experimental Mr (expt) masses [delta (Da)], number of missed trypsin cleavage sites (Miss) and peptide sequences (Peptide). The dots indicate trypsin cleavage sites. **(C)** SDS-PAGE (10% separation gel) (left) and corresponding PAS staining (right) of surface proteins from *S*. *aureus* 1061 (lane 2), *S*. *aureus* 1061*pls* (lane 3), and *S*. *aureus* 1061*pls* (pPLS4) (lane 4).

To identify glycosylated proteins in *S*. *aureus*, surface proteins from the MRSA strains COL and 1061 were analyzed (strains are listed in Table 1). Covalently linked surface proteins were prepared from cultures grown to exponential or stationary growth phase by lysostaphin treatment. Subsequently, the proteins were separated by SDS-PAGE and glycosylated proteins were detected by periodic acid-Schiff’s (PAS) staining. In the strain *S*. *aureus* COL, four glycosylated surface proteins with molecular masses of approximately >300, 250, 165, and 120 kDa were detected in lysostaphin lysates from overnight-grown cultures (Fig 1AI). Protein bands with the same molecular masses were present in lysostaphin lysates from *S*. *aureus* COL cultures grown to the exponential growth phase although to a lesser extent (1A II). In the strain *S*. *aureus* 1061, only two glycosylated surface proteins with molecular masses of >300 and ∼175 kDa were identified in lysostaphin lysates from overnight-grown cultures (Fig 1A). For comparison, no glycosylated surface protein could be detected in lysostaphin lysates from the apathogenic strain *Staphylococcus carnosus* TM300 (Fig 1A). No additional glycosylated proteins could also be detected from the preparations of surface-associated proteins of the strain COL (Fig 1AIII).

**Table 1:** Bacterial strains used in this study.

Strains and plasmids	Genotype or description^a^	Source
***S*.* aureus***		
COL	Clinical MRSA expressing *pls*	[27]
COL*sdgA/sdgB*	Deficient in *sdgA/sdgB* (Kan^r^)	This study
1061	Clinical MRSA expressing *pls*	[28]
1061*pls*	Deficient in *pls* (Tc^r^)	[26]
1061*pls* (pPLS4)	Complemented *pls* mutant (Tc^r^ Cm^r^)	[26]
1061*pls* (pPLS6)	Deletion of SD repeats of Pls (Tc^r^ Cm^r^)	[29]
1061*pls* (pPLSsub1)	Production of Pls with 17 aa of SD repeats (Tc^r^ Cm^r^)	This study
1061*pls* (pPLSsub2)	Production of Pls with 34 aa of SD repeats (Tc^r^ Cm^r^)	This study
1061*pls* (pPLSsub3)	Production of Pls with 130 aa of SD repeats (Tc^r^ Cm^r^)	This study
SA113	ATCC 35556, NCTC 8325 derivative, *rsbU*^-^	[30]
SA113 (pCU1)	Empty vector control (Cm^r^)	This study
SA113 (pPLS4)	Expression of *pls* (Cm^r^)	This study
SA113*gtfA* (pPLS4)	Deficient in *gtfA*, expression of *pls* (Cm^r^)	This study
SA113*bgt* (pPLS4)	Deficient in the putative *bgt*, expression of *pls* (Cm^r^)	This study
SA113*gtfA*/*sdgA/sdgB* (pPLS4)	Deficient in *gtfA*, *sdgA*, *sdgB*, expression of *pls* (Em^r^ Cm^r^)	This study
SA113*sdgA/sdgB*	Deficient in *sdgA*, *sdgB* (Em^r^)	This study
SA113*sdgA/sdgB* (pCU1)	Empty vector control (Em^r^ Cm^r^)	This study
SA113*sdgA/sdgB* (pPLS4)	Expression of *pls* (Em^r^ Cm^r^)	This study
SA113*sdgA/sdgB* (pPlsGtfCD_COL_)	Expression of *pls*, *gtfC*, *gtfD* from strain COL (Em^r^ Cm^r^)	This study
SA113*sdgA/sdgB* (pPlsGtfΔCD_COL_)	Expression of *pls*, Δ*gtfC*, *gtfD* from strain COL (Em^r^ Cm^r^)	This study
SA113*sdgA/sdgB* (pPlsGtfCΔD_COL_)	Expression of *pls*, *gtfC*, Δ*gtfD* from strain COL (Em^r^ Cm^r^)	This study
SA113*sdgA/sdgB* (pPlsGtfΔCΔD_COL_)	Expression of *pls*, Δ*gtfC*, Δ*gtfD* from strain COL (Em^r^ Cm^r^)	This study
SA113*sdgA/sdgB* (pPlsGtfCD_1061_)	Expression of *pls*, *gtfC*, *gtfD* from strain 1061 (Em^r^ Cm^r^)	This study
SA113*sdgA/sdgB* (pPlsGtfΔCD_1061_)	Expression of *pls*, Δ*gtfC*, *gtfD* from strain 1061 (Em^r^ Cm^r^)	This study
SA113*sdgA/sdgB* (pPlsGtfCΔD_1061_)	Expression of *pls*, *gtfC*, Δ*gtfD* from strain 1061 (Em^r^ Cm^r^)	This study
SA113*sdgA/sdgB* (pPlsGtfΔCΔD_1061_)	Expression of *pls*, Δ*gtfC*, Δ*gtfD* from strain 1061 (Em^r^ Cm^r^)	This study
SH1000	ATCC 35556, NCTC 8325 derivative, *rsbU*^+^	[31]
SH1000 (pPLS4)	Expression of *pls* (Cm^r^)	This study
SH1000*sdgA/sdgB*	Deficient in *sdgA/sdgB* (Kan^r^)	This study
SH1000*sdgA/sdgB* (pPLS4)	Expression of *pls* (Kan^r^ Cm^r^)	This study
Newman	NCTC 8178, clinical isolate	[32]
Newman (pPLS4)	Expression of *pls* (Cm^r^)	This study
Newman (pCU1)	Empty vector control (Em^r^ Cm^r^)	This study
Newman (pPlsGtfCD_COL_)	Expression of *pls*, *gtfC*, *gtfD* from strain COL (Em^r^ Cm^r^)	This study
Newman (pPlsGtfΔCΔD_COL_)	Expression of *pls*, Δ*gtfC*, Δ*gtfD* from strain COL (Em^r^ Cm^r^)	This study
Newman (pΔPlsGtfCD_COL_)	Expression of Δ*pls*, *gtfC*, *gtfD* from strain COL (Em^r^ Cm^r^)	This study
Newman_C_	Newman (pPlsGtfCD_COL_) cured from its plasmid	This study
Newman_C_ (pCU1)	Newman_C_ transformed with plasmid pCU1	This study
Newman*sdgA/sdgB*	Deficient in *sdgA/sdgB* (Kan^r^)	This study
Newman*sdgA/sdgB* (pPLS4)	Expression of *pls* (Kan^r^ Cm^r^)	This study
Newman*sdgA/sdgB* (pCU1)	Empty vector control (Em^r^ Cm^r^)	This study
Newman*sdgA/sdgB* (pPlsGtfCD_COL_)	Expression of *pls*, *gtfC*, *gtfD* from strain COL (Em^r^ Cm^r^)	This study
Newman*sdgA/sdgB* (pPlsGtfΔCΔD_COL_)	Expression of *pls*, Δ*gtfC*, Δ*gtfD* from strain COL (Em^r^ Cm^r^)	This study
Newman*sdgA/sdgB* (pΔPlsGtfCD_COL_)	Expression of Δ*pls*, *gtfC*, *gtfD* from strain COL (Em^r^ Cm^r^)	This study
Newman*sdgA/sdgB*_C_	Newman*sdgA/sdgB* (pPlsGtfCD_COL_) cured from its plasmid	This study
Newman*sdgA/sdgB*_C_ (pCU1)	Newman*sdgA/sdgB*_C_ transformed with plasmid pCU1	This study
Cowan 1	ATCC 12598, NCTC 8530, isolate from septic arthritis	[32]
***S*. *carnosus***		
TM300	Non-pathogenic reference isolate	[33]
TM300 (pCU1)	Empty vector control (Cm^r^)	This study
TM300 (pPlsGtfCD_COL_)	Expression of *pls*, *gtfC*, *gtfD* from strain COL (Em^r^ Cm^r^)	This study
TM300 (pPlsGtfΔCD_COL_)	Expression of *pls*, Δ*gtfC*, *gtfD* from strain COL (Em^r^ Cm^r^)	This study
TM300 (pPlsGtfCΔD_COL_)	Expression of *pls*, *gtfC*, Δ*gtfD* from strain COL (Em^r^ Cm^r^)	This study
TM300 (pPlsGtfΔCΔD_COL_)	Expression of *pls*, Δ*gtfC*, Δ*gtfD* from strain COL (Em^r^ Cm^r^)	This study
TM300 (pPlsGtfCD_1061_)	Expression of *pls*, *gtfC*, *gtfD* from strain 1061 (Em^r^ Cm^r^)	This study
TM300 (pPlsGtfΔCD_1061_)	Expression of *pls*, Δ*gtfC*, *gtfD* from strain 1061 (Em^r^ Cm^r^)	This study
TM300 (pPlsGtfCΔD_1061_)	Expression of *pls*, *gtfC*, Δ*gtfD* from strain 1061 (Em^r^ Cm^r^)	This study
TM300 (pPlsGtfΔCΔD_1061_)	Expression of *pls*, Δ*gtfC*, Δ*gtfD* from strain 1061 (Em^r^ Cm^r^)	This study
***S*. *epidermidis***		
RP62A	Strong biofilm producer	[34]
***E*. *coli***		
XL1-Blue	*supE44 hsdR17 recA1 endA1 gyrA46 thi relA1 lac* F’[*proAB*^*+*^ *lacI*^*q*^ *lacZ*Δ*M15* Tn*10*] (Tc^r^), cloning host	[35]

^a^MRSA, methicillin-resistant *S*. *aureus*; *gtf*, glycosyltransferase; *bgt*, bactoprenol glycosyltransferase, *pls*, plasmin-sensitive protein; Tc^r^, tetracycline resistant; Cm^r^, chloramphenicol resistant; Em^r^, erythromycin resistant; Kan^r^, kanamycin resistant.

### The *S*. *aureus* plasmin-sensitive protein Pls is a glycosylated protein

To identify the glycosylated proteins, the ∼250-kDa and ∼165-kDa proteins from strain COL and the ∼175-KDa protein from strain 1061 were excised and subjected to mass spectrometry (MS). All three proteins were identified as the plasmin-sensitive protein Pls (Fig 1B). Pls is a covalently cell wall-anchored protein of MRSA strains with a reported apparent molecular mass of 230 kDa that is sensitive to proteolysis by plasmin leading to 175-kDa and 68-kDa cleavage products [26]. The predicted Pls polypeptide from strain COL has 1,548 aa and a calculated molecular mass of 165 kDa and thus is slightly smaller than Pls from strain 1061, which consists of 1,637 aa and has a calculated molecular mass of 175 kDa. Thus, the reported apparent molecular mass of Pls is much higher than the calculated molecular mass [26], which at least in part might be due to its glycosylation.

To verify that Pls is a glycosylated protein, we analyzed the surface proteins prepared from the *pls* mutant strain *S*. *aureus* 1061*pls* and the complemented mutant *S*. *aureus* 1061*pls* (pPLS4) by PAS staining (Fig 1C). The previously reported plasmid pPLS4 [26] encodes the *pls* gene from strain 1061 (see below, Fig 5A). The ∼175-kDa glycosylated surface protein was missing from the 1061*pls* mutant strain (lane 3), but present in the wild-type strain 1061 (lane 2) and the complemented mutant strain (lane 4) confirming that Pls is a glycosylated protein (Fig 1C).

### Identification of the Gtfs mediating glycosylation of Pls

#### Expression of *pls* in *S*. *aureus gtf* mutant strains

**Fig 2:**
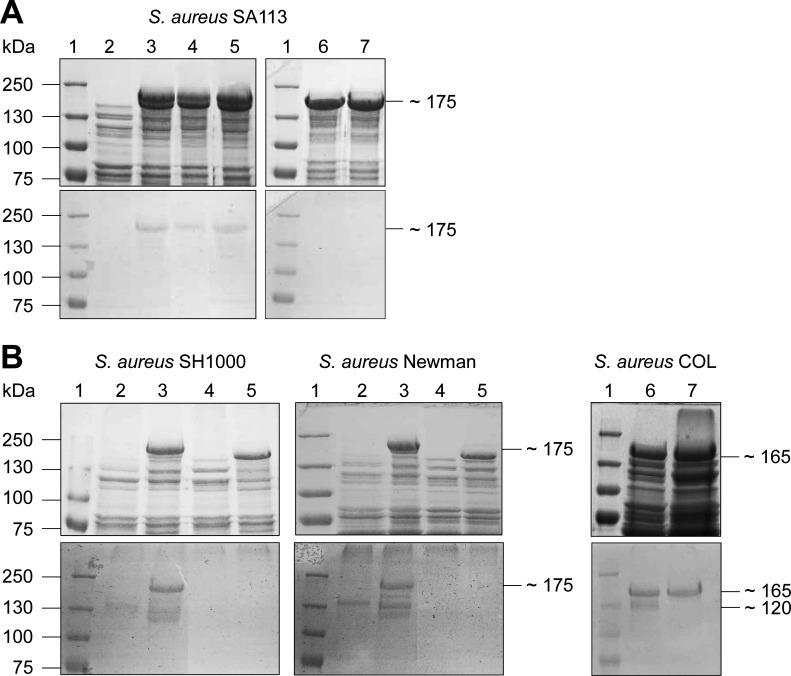
The glycosyltransferases SdgA/SdgB are involved in the glycosylation of Pls. **(A)** SDS-PAGE (7.5% separation gel) (upper panel) and corresponding PAS staining (lower panel) of surface proteins from *S*. *aureus* SA113 (lane 2), SA113 (pPLS4) (lane 3), SA113*bgt* (pPLS4) (lane 4), SA113*gtfA* (pPLS4) (lane 5), SA113*sdgA/sdgB* (pPLS4) (lane 6), and SA113*gtfA*/*sdgA/sdgB* (pPLS4) (lane 7). Glycosylated Pls is only produced when SdgA/SdgB are present. **(B)** SDS-PAGE (7.5% separation gel) (upper panel) and corresponding PAS staining (lower panel) of surface proteins from *S*. *aureus* SH1000 (lane 2), SH1000 (pPLS4) (lane 3), SH1000*sdgA/sdgB* (lane 4) SH1000*sdgA/sdgB* (pPLS4) (lane 5), *S*. *aureus* Newman (lane 2), Newman (pPLS4) (lane 3), Newman*sdgA/sdgB* (lane 4) Newman*sdgA/sdgB* (pPLS4) (lane 5), *S*. *aureus* COL (lane 6), COL*sdgA/sdgB* (lane 7). Both, *S*. *aureus* COL and *S*. *aureus* COL*sdgA/sdgB* produce glycosylated Pls.

Carbohydrates are transferred to the target proteins by Gtfs. To our knowledge, so far only four Gtfs from *S*. *aureus*, GtfA/GtfB and SdgA/SdgB, have been associated with the glycosylation of staphylococcal surface proteins, i.e. the platelet-binding protein SraP and the clumping factor ClfA, respectively [15, 16, 20]. To identify the Gtfs involved in the glycosylation of Pls, we expressed the *pls* gene encoded on plasmid pPLS4 [26] (see also below) in different *gtf* mutants previously constructed from strain *S*. *aureus* SA113 (SA113*gtfA*, SA113*sdgA/sdgB*, SA113*gtfA*/*sdgA/sdgB*, SA113*bgt*) and analyzed the surface proteins produced by the respective strains for their glycosylation (Fig 2A). SDS-PAGE revealed that the strains SA113 (pPLS4) (lane 3), SA113*bgt* (pPLS4) (lane 4), and SA113*gtfA* (pPLS4) (lane 5) all produced two additional large surface proteins that were not produced by the negative control SA113 (lane 2) (Fig 2A, upper panel) with the larger protein being glycosylated as detected by PAS staining (Fig 2A, lower panel). In contrast, the strains SA113*sdgA/sdgB* (pPLS4) (lane 6) and SA113*gtfA*/*sdgA/sdgB* (pPLS4) (lane 7) each additionally produced only one large protein (Fig 2A, upper panel), which was not glycosylated (Fig 2A, lower panel) suggesting that *sdgA/sdgB* mediate glycosylation of Pls. To further verify the importance of *sdgA/sdgB* in the glycosylation of Pls, we constructed *sdgA/sdgB* knockout mutants also from the strains SH1000 and Newman and expressed *pls* in the respective mutants. While the wild-type strains SH1000 (pPLS4) (lane 3) and Newman (pPLS4) (lane 3) produced a glycosylated protein with a molecular mass of ∼175 kDa corresponding to Pls as determined by PAS staining (Fig 2B, lower panel), the mutant strains SH1000*sdgA/sdgB* (pPLS4) (lane 5) and Newman*sdgA/sdgB* (pPLS4) (lane 5) produced only a non-glycosylated version of Pls (Fig 2B, upper and lower panel). For comparison, the negative controls SH1000 (lane 2), SH1000*sdgA/sdgB* (lane 4), Newman (lane 2), and Newman*sdgA/sdgB* (lane 4) did not produce surface proteins with the respective molecular masses (Fig 2B, upper and lower panel). To analyze, whether the homologous SdgA/SdgB from the MRSA strain COL also mediate glycosylation of Pls, we constructed a COL*sdgA/sdgB* mutant. PAS staining revealed glycosylated surface proteins with molecular masses of ∼165 kDa and ∼120 kDa in lysostaphin lysates from strain COL (Fig 2B, lower panel, lane 6), the latter being absent from lysostaphin lysates from its *sdgA/sdgB* mutant (Fig 2B, lower panel, lane 7). MS confirmed that the ∼165-kDa glycosylated protein produced by strain COL*sdgA/sdgB* is Pls (S1 Fig). These results clearly demonstrate that SdgA/SdgB are involved in the glycosylation of Pls, when produced by various methicillin-sensitive *S*. *aureus* (MSSA) strains and that different or additional Gtfs are involved in the glycosylation of Pls in strain COL.

#### Identification of the genes *gtfC* and *gtfD* involved in glycosylation of Pls in the MRSA strains COL and 1061

**Fig 3:**
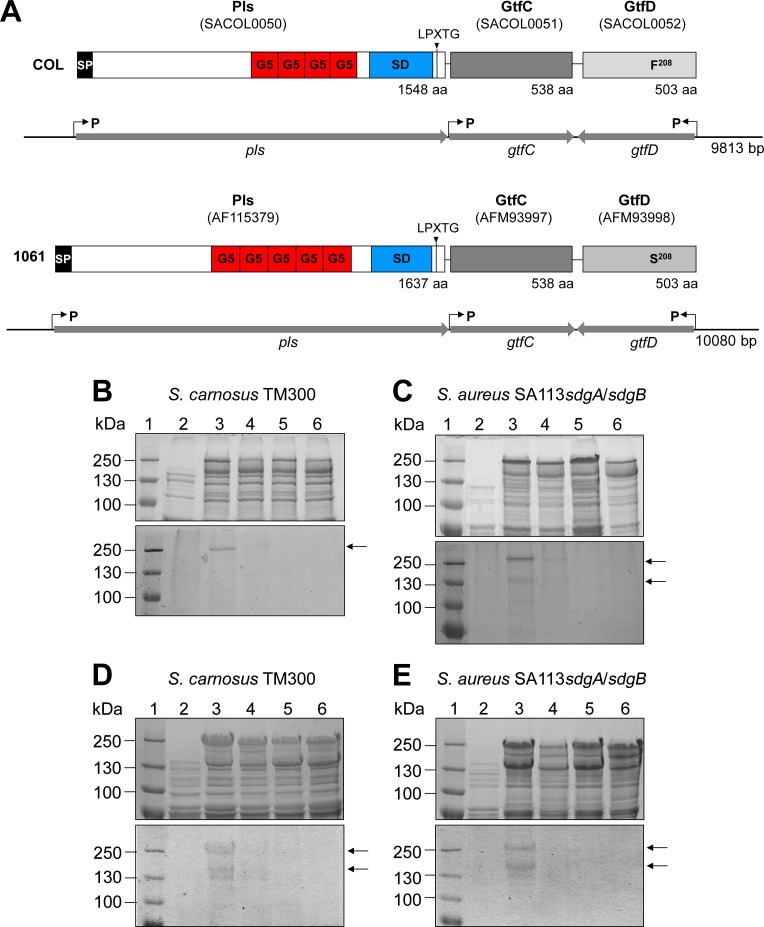
The glycosyltransferases GtfC/GtfD mediate glycosylation of Pls. **(A)** Schematic model of the 9.8 kbp and 10 kbp DNA fragment expressing *pls*, *gtfC*, and *gtfD* and the corresponding gene products Pls, GtfC, and GtfD from strain COL and strain 1061, respectively. The *gtfC* and *gtfD* genes are located downstream of *pls* and convergently transcribed. Putative promotors are indicated by arrows. SD; SD-repeat region, G5; G5 domains (Pfam accession number: PF077501), LPXTG; C-terminal cell-wall anchor motif. **(B, C)** SDS-PAGE (10% separation gel) (upper panels) and corresponding PAS staining (lower panels) of surface proteins from *S*. *carnosus* TM300 **(B)** and *S*. *aureus* SA113*sdgA/sdgB* **(C)** strains expressing genes from strain COL. *S*. *carnosus* TM300 and *S*. *aureus* SA113*sdgA/sdgB* produce glycosylated versions of Pls when *pls* is coexpressed with *gtfC*/*gtfD*. The lanes contain: **(B)** 2; *S*. *carnosus* (pCU1), 3; *S*. *carnosus* (pPlsGtfCD_COL_), 4; *S*. *carnosus* (pPlsGtfΔCD_COL_), 5; *S*. *carnosus* (pPlsGtfCΔD_COL_), 6; *S*. *carnosus* (pPlsGtfΔCΔD_COL_). **(C)** 2; *S*. *aureus* SA113*sdgA/sdgB* (pCU1), 3; *S*. *aureus* SA113*sdgA/sdgB* (pPlsGtfCD_COL_), 4; *S*. *aureus* SA113*sdgA/sdgB* (pPlsGtfΔCD_COL_), 5; *S*. *aureus* SA113*sdgA/sdgB* (pPlsGtfCΔD_COL_), 6; *S*. *aureus* SA113*sdgA/sdgB* (pPlsGtfΔCΔD_COL_). The sizes of the marker proteins (1; kDa) are indicated on the left. **(D, E)** SDS-PAGE (10% separation gel) (upper panels) and corresponding PAS staining (lower panels) of surface proteins from *S*. *carnosus* TM300 **(D)** and *S*. *aureus* SA113*sdgA/sdgB* **(E)** strains expressing genes from strain 1061. *S*. *carnosus* TM300 and *S*. *aureus* SA113*sdgA/sdgB* produce glycosylated versions of Pls when *pls* is coexpressed with *gtfC*/*gtfD*. The lanes contained: **(D)** 2; *S*. *carnosus* (pCU1), 3; *S*. *carnosus* (pPlsGtfCD_1061_), 4; *S*. *carnosus* (pPlsGtfΔCD_1061_), 5; *S*. *carnosus* (pPlsGtfCΔD_1061_), 6; *S*. *carnosus* (pPlsGtfΔCΔD_1061_). **(E)** 2; *S*. *aureus* SA113*sdgA/sdgB* (pCU1), 3; *S*. *aureus* SA113*sdgA/sdgB* (pPlsGtfCD_1061_), 4; *S*. *aureus* SA113*sdgA/sdgB* (pPlsGtfΔCD_1061_), 5; *S*. *aureus* SA113*sdgA/sdgB* (pPlsGtfCΔD_1061_), 6; *S*. *aureus* SA113*sdgA/sdgB* (pPlsGtfΔCΔD_1061_). The sizes of the marker proteins (1; kDa) are indicated on the left.

In a search for further potential Gtfs mediating glycosylation of Pls, we analyzed the DNA sequences adjacent to the *pls* structural gene (SACOL0050) by the Carbohydrate-Active enZymes (CAZy) database [36] that predicts 21 Gtfs encoded by the genome of *S*. *aureus* COL. We identified two putative *gtf* genes encoded downstream of *pls* on the SCC*mec* element that are transcribed convergently (Fig 3A). The 503-aa SACOL0052 (termed GtfD) was predicted to be a Gtf by the CAZy database. The 538-aa SACOL0051 (termed GtfC) encoded immediately downstream of *pls* was annotated as a hypothetical protein and not predicted to be a Gtf by the CAZy database. However, our BlastP search revealed a very high similarity (92% identical aa) with a putative α-glycosyltransferase from *S*. *aureus* C75S and 69% identical aa with a putative α-glycosyltransferase from *S*. *epidermidis* ATCC 12228.

To test our hypothesis that GtfC and/or GtfD are involved in the glycosylation of Pls, we generated a *pls* expression clone from *S*. *aureus* COL that contained 902 bp upstream of the *pls* gene and 833 bp upstream of the *gtfD* gene and expressed *pls* together with *gtfC* and *gtfD* under the control of their putative natural promoters creating plasmid pPlsGtfCD_COL_ in the cloning host *S*. *carnosus* TM300 (Fig 3B). This strain is suitable as cloning host as it did not produce glycosylated surface proteins (Fig 3B, lane 2) and does not contain genes homologous to *gtfC*/*gtfD* according to our BlastP searches. Moreover, *pls* expression clones were constructed from this clone with a site-directed mutation in either *gtfC* (creating plasmid pPlsGtfΔCD_COL_), *gtfD* (creating plasmid pPlsGtfCΔD_COL_), or in both genes, *gtfC*/*gtfD* (creating plasmid pPlsGtfΔCΔD_COL_). SDS-PAGE (upper panel) and corresponding PAS staining (lower panel) revealed that *S*. *carnosus* produced a glycosylated version of Pls, when both genes downstream of *pls*, *gtfC* and *gtfD*, are coexpressed with *pls* [Fig 3B, *S*. *carnosus* (pPlsGtfCD_COL_) (lane 3; visible as ∼250-kDa protein band in the lower panel)]. In contrast, *S*. *carnosus* produces a non-glycosylated version of Pls, when *gtfC* and *gtfD* or only *gtfD* were deleted [Fig 3B, *S*. *carnosus* (pPlsGtfΔCΔD_COL_) (lane 6), *S*. *carnosus* (pPlsGtfCΔD_COL_) (lane 5)]. A weakly glycosylated version of Pls was detected by PAS staining, when *pls* was coexpressed with the intact *gtfD* [Fig 3B, lower panel, *S*. *carnosus* (pPlsGtfΔCD_COL_) (lane 4)].

To analyze the glycosylation of Pls also in the *S*. *aureus* background, we expressed the respective plasmids in the strain SA113*sdgA/sdgB*. PAS staining (lower panel) revealed that the SA113*sdgA/sdgB* mutant did not produce a glycosylated version of Pls, when *gtfC* and *gtfD* or only *gtfD*, were deleted [Fig 3C, SA113*sdgA/sdgB* (pPlsGtfΔCΔD_COL_) (lane 6), SA113*sdgA/sdgB* (pPlsGtfCΔD_COL_) (lane 5)], although corresponding SDS-PAGE (upper panel) demonstrated that the corresponding Pls proteins were produced. Like with *S*. *carnosus*, SDS-PAGE (upper panel) and corresponding PAS staining (lower panel) revealed the production of a glycosylated version of Pls by the SA113*sdgA/sdgB* mutant, when both genes, *gtfC* and *gtfD*, are coexpressed with *pls* [Fig 3C, SA113*sdgA/sdgB* (pPlsGtfCD_COL_) (lane 3; visible as ∼250-kDa and ∼165-kDa protein bands in the lower panel)]. Furthermore as in *S*. *carnosus*, the presence of the intact *gtfD* in SA113*sdgA/sdgB* is sufficient to produce a weakly glycosylated version of Pls [Fig 3C, lower panel, SA113*sdgA/sdgB* (pPlsGtfΔCD_COL_) (lane 4)]. Thus, GtfD might be sufficient for an initial glycosylation of Pls, but both, GtfC and GtfD seem to be required for the production of the fully glycosylated version of Pls.

Analogously, respective *pls* and *gtfC*/*gtfD* expression clones were constructed from chromosomal DNA from strain 1061 (Fig 3A) and analyzed (Fig 3D and 3E). SDS-PAGE (upper panel) and PAS staining (lower panel) revealed that *S*. *carnosus* (pPlsGtfCD_1061_) (Fig 3D, lane 3) and SA113*sdgA/sdgB* (pPlsGtfCD_1061_) (Fig 3E, lane 3) produced a glycosylated version of Pls as expected. Furthermore, the strains *S*. *carnosus* (pPlsGtfΔCΔD_1061_) (Fig 3D, lane 6), SA113*sdgA/sdgB* (pPlsGtfΔCΔD_1061_) (Fig 3E, lane 6), *S*. *carnosus* (pPlsGtfCΔD_1061_) (Fig 3D, lane 5), and SA113*sdgA/sdgB* (pPlsGtfCΔD_1061_) (Fig 3E, lane 5) did not produce a glycosylated version of Pls as expected. However surprisingly, in contrast to the respective clones expressing the genes from strain COL, the strains *S*. *carnosus* (pPlsGtfΔCD_1061_) (Fig 3D, lane 4) and SA113*sdgA/sdgB* (pPlsGtfΔCD_1061_) (Fig 3E, lane 4) did not produce a glycosylated version of Pls suggesting that GtfD from strain 1061 is not sufficient for an initial glycosylation of Pls and requires the additional activity of GtfC.

### Purification of glycosylated Pls by using the lectin concanavalin A (ConA)

**Fig 4:**
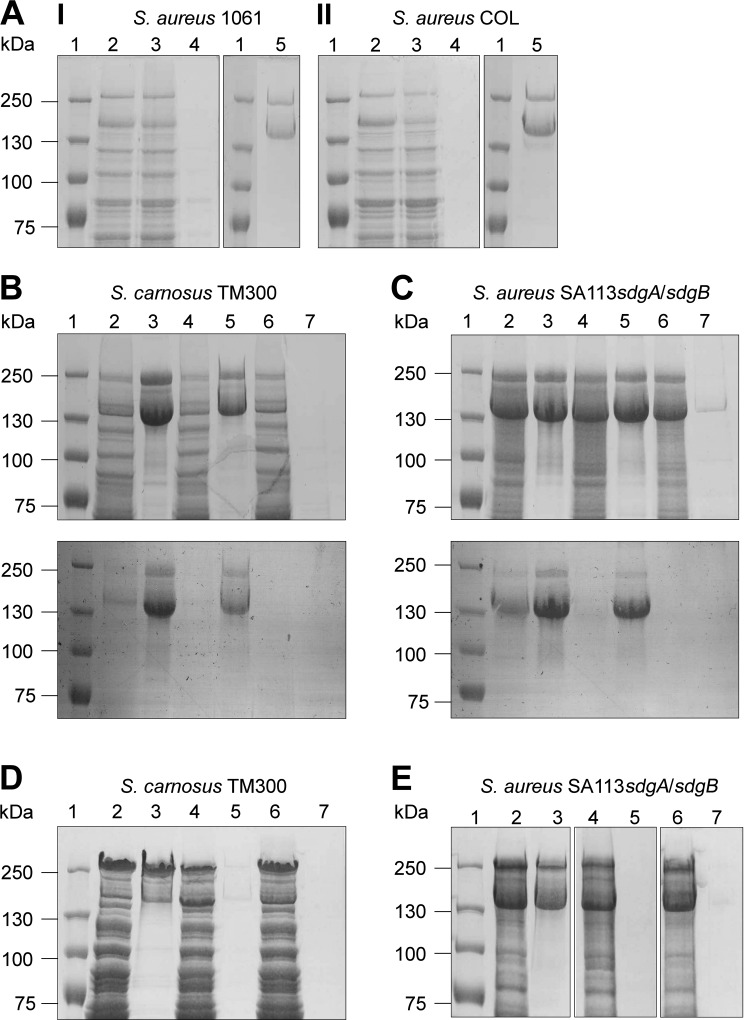
Purification of Pls by using ConA. (**A**) SDS-PAGE (7.5% separation gel) of surface proteins from *S*. *aureus* 1061 (**I**) and COL (**II**) and different fractions collected from a column packed with ConA sepharose. The lanes contained: 2; lysate fraction, 3; flow-through fraction, 4; wash fraction, 5; eluted fraction. The sizes of the marker proteins (lane 1; kDa) are indicated on the left. (**B-E**) Purification of Pls produced by *S*. *carnosus* TM300 (**B, D**) or by *S*. *aureus* SA113*sdgA/sdgB* (**C, E**) by using ConA. (**B, C**) SDS-PAGE (7.5% separation gel) (upper panel) and corresponding PAS staining (lower panel) and (**D, E**) SDS-PAGE (7.5% separation gel) of surface proteins (lanes 2, 4, 6) and eluted fractions (lanes 3, 5, 7) collected from a column packed with ConA sepharose. The lanes contained: (**B**) 2,3: *S*. *carnosus* (pPlsGtfCD_COL_); 4,5: *S*. *carnosus* (pPlsGtfΔCD_COL_); 6,7; *S*. *carnosus* (pPlsGtfCΔD_COL_). (**D**) 2,3: *S*. *carnosus* (pPlsGtfCD_1061_); 4,5: *S*. *carnosus* (pPlsGtfΔCD_1061_); 6,7; *S*. *carnosus* (pPlsGtfCΔD_1061_). (**C**) 2,3: *S*. *aureus* SA113*sdgA/sdgB* (pPlsGtfCD_COL_); 4,5: *S*. *aureus* SA113*sdgA/sdgB* (pPlsGtfΔCD_COL_); 6,7: *S*. *aureus* SA113*sdgA/sdgB* (pPlsGtfCΔD_COL_). (**E**) 2,3: *S*. *aureus* SA113*sdgA/sdgB* (pPlsGtfCD_1061_); 4,5: *S*. *aureus* SA113*sdgA/sdgB* (pPlsGtfΔCD_1061_); 6,7: *S*. *aureus* SA113*sdgA/sdgB* (pPlsGtfCΔD_1061_). The sizes of the marker proteins (lane 1; kDa) are indicated on the left.

To verify these observations and to exclude the possibility that glycosylated Pls produced by the strains *S*. *carnosus* (pPlsGtfΔCD_1061_) and SA113*sdgA/sdgB* (pPlsGtfΔCD_1061_) is below the detection limit, we purified the respective glycosylated proteins by using the lectin ConA. Lectins are carbohydrate-binding proteins that have high substrate specificity [37–39]. It has been reported before that Pls can be purified by using the lectin WGA [28]. Here, we successfully purified Pls from the strains 1061 (Fig 4AI, lane 5) and COL (Fig 4AII, lane 5) by using ConA. Moreover, we could purify Pls by using ConA, when heterologously expressed by *S*. *carnosus* TM300 (pPlsGtfCD_COL_) (Fig 4B, lane 3), *S*. *carnosus* TM300 (pPlsGtfCD_1061_) (Fig 4D, lane 3), SA113*sdgA/sdgB* (pPlsGtfCD_COL_) (Fig 4C, lane 3) or SA113*sdgA/sdgB* (pPlsGtfCD_1061_) (Fig 4E, lane 3). Pls could also be purified by using ConA from *S*. *carnosus* TM300 (pPlsGtfΔCD_COL_), when only *gtfD* was coexpressed with *pls* (Fig 4B, lane 5). In contrast, Pls could not be purified from strain *S*. *carnosus* TM300 (pPlsGtfCΔD_COL_) (Fig 4B, lane 7) confirming our results presented in Fig 3B that suggested that GtfD is necessary for an initial glycosylation of Pls and GtfC is involved in further glycosylation, but dispensable. Very similar results were obtained, when Pls was purified from SA113*sdgA/sdgB* (pPlsGtfΔCD_COL_) (Fig 4C, lane 5) and from SA113*sdgA/sdgB* (pPlsGtfCΔD_COL_) (Fig 4C, lane 7). As expected, it was not possible to purify Pls from the strains *S*. *carnosus* TM300 (pPlsGtfCΔD_1061_) (Fig 4D, lane 7) and SA113*sdgA/sdgB* (pPlsGtfCΔD_1061_) (Fig 4E, lane 7). In agreement with our results from Fig 3D and 3E, it was not possible to purify Pls from strains *S*. *carnosus* TM300 (pPlsGtfΔCD_1061_) (Fig 4D, lane 5) and SA113*sdgA/sdgB* (pPlsGtfΔCD_1061_) (Fig 4E, lane 5) by using ConA thus confirming that GtfD from strain 1061 unlike that from strain COL does not seem to be sufficient for an initial glycosylation of Pls and requires the additional activity of GtfC.

### The serine aspartate (SD)-repeat region of Pls is glycosylated

**Fig 5:**
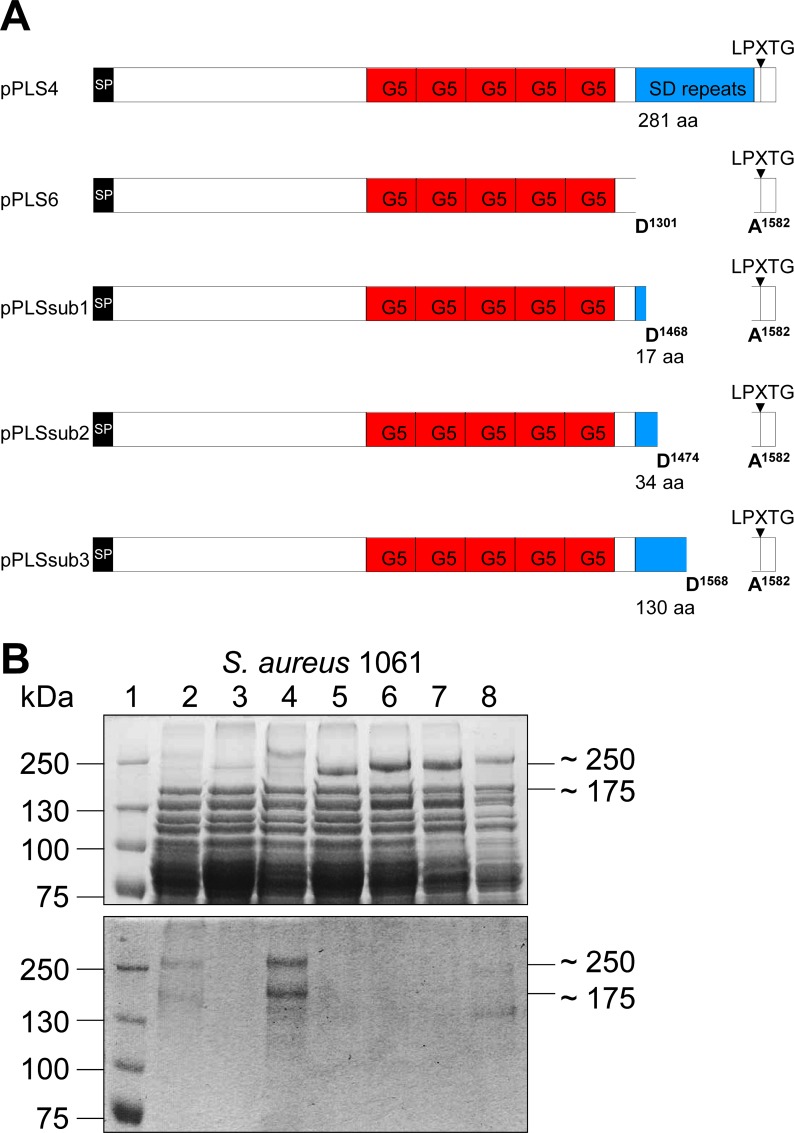
The SD-repeat region of Pls is glycosylated. **(A)** Schematic map of Pls from strain 1061 and its truncated derivatives encoded by the indicated plasmids. SD; SD-repeat region, G5; G5 domains, LPXTG; C-terminal cell-wall anchor motif. **(B)** SDS-PAGE (7.5% separation gel) (upper panel) and corresponding PAS staining (lower panel) of surface proteins from the strains *S*. *aureus* 1061 (lane 2), 1061*pls* (lane 3), 1061*pls* (pPLS4) (lane 4), 1061*pls* (pPLS6) (lane 5), 1061*pls* (pPLSsub1) (lane 6), 1061*pls* (pPLSsub2) (lane 7), 1061*pls* (pPLSsub3) (lane 8). The sizes of the marker proteins (lane 1; kDa) are indicated on the left.

In order to identify the region of glycosylation in Pls, we transformed the strain 1061*pls* with the plasmid pPLS6 that encodes the *pls* gene with a deleted SD-repeat region [29] (Fig 5A). Furthermore, we generated different subclones from plasmid pPLS4 in strain 1061*pls* that led to the production of truncated versions of Pls with 17 aa (pPLSsub1), 34 aa (pPLSsub2), or 130 aa (pPLSsub3) of the SD-repeat region (Fig 5A). SDS-PAGE of surface proteins revealed that the 1061*pls* strains harboring the plasmids pPLS4 (lane 4), pPLS6 (lane 5), pPLSsub1 (lane 6), pPLSsub2 (lane 7), or pPLSsub3 (lane 8) all produced a large surface protein with the expected molecular masses in contrast to the control strain 1061*pls* (lane 3) (Fig 5B, upper panel). However, PAS staining revealed that only the strains 1061*pls* (pPLS4) (lane 4) and 1061*pls* (pPLSsub3) (lane 8) produced a glycosylated version of Pls (Fig 5B, lower panel). This indicates that the SD-repeat region of Pls is modified by glycosyl residues with an apparent minimal requirement of > 34 aa of the SD-repeat region. The intensity of the glycostained protein bands produced by strain 1061*pls* (pPLSsub3) (lane 8) is markedly decreased in comparison to that produced by strain 1061*pls* (pPLS4) (lane 4) strongly suggesting a lower number of attached sugar moieties due to the shortened SD-repeat region with strain 1061*pls* (pPLSsub3).

### Determination of modifying glycosyl residues

**Fig 6:**
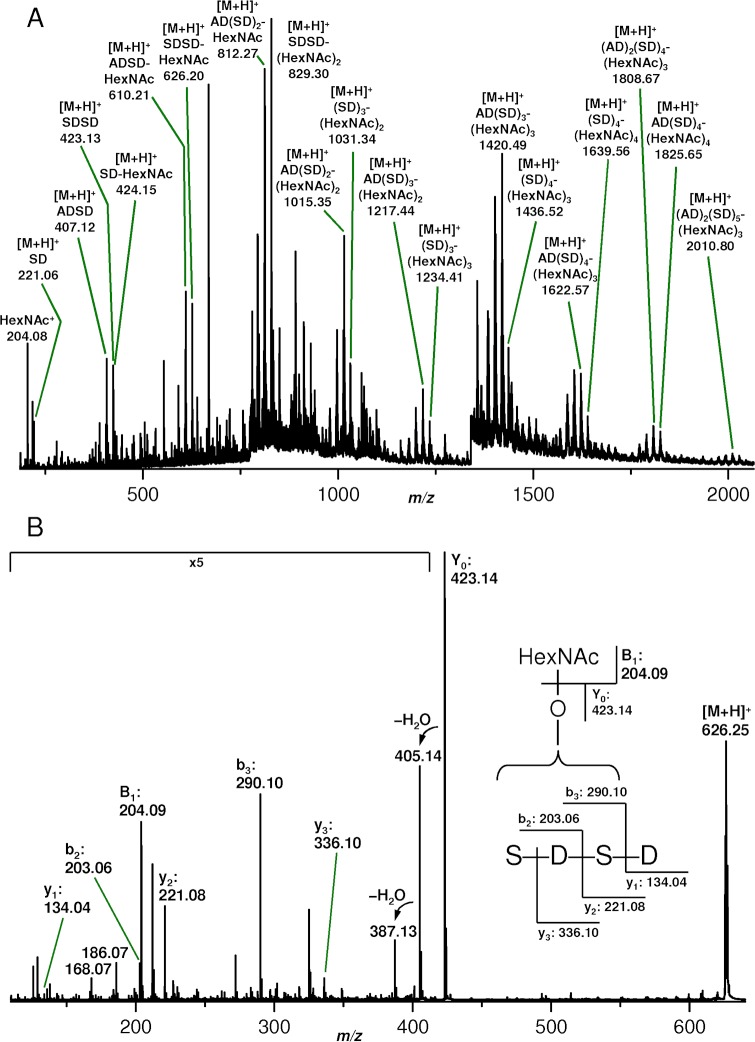
Determination of the modifying carbohydrate moieties. **(A)** NanoESI Q-Tof mass spectrum of a hydrolysate obtained from a Pls preparation derived from *S*. *aureus* strain 1061 by incubation with 12.5% (v/v) acetic acid for 2 h at 95°C. For reasons of clarity only a few signals originating from (glyco)peptides derived from the SD repeats are labeled. A summary of all detected corresponding species is given in S1 Table. **(B)** NanoESI Q-Tof fragment ion spectrum obtained from a CID experiment on the singly charged precursor glycopeptide ions with *m/z* 626.25. The insert shows the corresponding fragmentation scheme.

Pls preparations from the strains COL and 1061 were extensively digested by use of trypsin, chymotrypsin, endoproteinase Glu-C, and thermolysin and the proteolytic peptides were subsequently analyzed and sequenced by means of MS. Though leading to high sequence coverages of the non-SD repeat regions of the proteins, no hint on glycosylation was obtained. This further confirmed that glycosylation was restricted to the SD repeats that were, however, not susceptible to proteolysis. Even thermolysin (supposed to cleave N-terminal to alanine) and pronase (yielding randomly cleaved short peptides down to single aa) failed to produce (glyco)peptides derived from the SD repeats. Acid hydrolysis in the presence of 12.5% (v/v) acetic acid at 95°C was finally successful with respect to the preparation of the desired (glyco)peptides. The hydrolytic (glyco)peptides were analyzed by nanoESI MS and the spectrum resulting from the hydrolysate of Pls derived from strain 1061 is shown in Fig 6A. A number of species with aa compositions (SD)_n_ and (AD)_1-2_(SD)_m_ carrying 0-n and 0-m *N*-acetylhexosamine (HexNAc) moieties, respectively, as monosaccharides most probably attached to serine residues. For reasons of clarity only a few of them are labeled in the spectrum (Fig 6A), but a summary of all detected species is given in S1 Table. For a closer inspection, selected glycopeptides ion species were subjected to collision-induced dissociation (CID). The fragment ion spectra—an example ([M+H]^+^ of (SD)_2_-HexNAc) is shown in Fig 6B—corroborating the assumed structures and confirming that acid hydrolysis was achieved by cleavage of peptide bonds C-terminal to aspartic acid as has been reported earlier [40]. Moreover, isobaric species, i. e. sequence isomers in alanine-containing glycopeptides could be identified. However, the positions of the HexNAc residues could not be unambiguously determined since only very few glycosylated fragment ions were detectable. Similar results were obtained for Pls derived from strain COL. The large number of observed (glyco)peptides and the fact that peptides carry independently of their length from no up to maximum number of HexNAc moieties indicate that glycosylation as well as hydrolytic cleavage are random processes.

In order to get a clue whether SdgA/SdgB play a role in Pls glycosylation in MRSA strains, an estimation was made by comparing the ratios of the relative intensities of MS peaks corresponding to glycosylated and non-glycosylated SD repeat hydrolytic peptides obtained from Pls preparations of the *S*. *aureus* strain COL and its *sdgA/sdgB* mutant. The result is shown in Table 2. Indeed, the intensity ratios I_(SD)-HexNAc_/I_(SD)_, I_(SDAD)-HexNAc_/I_(SDAD)_, I_(SDSD)-HexNAc_/I_(SDSD)_, and I_(SDSD)-HexNAc2_/I_(SDSD)_ were lower by a factor of approximately 2 to 3 for hydrolysates of Pls from the COL*sdgA/sdgB* mutant compared to the wild-type Pls. This result gives some confirmation that SdgA/SdgB participate in the glycosylation of Pls.

**Table 2:** Ratios of relative intensities of signals derived from glycosylated and non-glycosylated SD-repeat hydrolytic peptides obtained from Pls preparations of *S*. *aureus* strain COL and its *sdgA/sdgB* mutant.

Peptide	SD	SD-HexNAc	SDAD	SDAD- HexNAc	SDSD	SDSD- HexNAc	SDSD- HexNAc_2_
Sample	*m/z*	*m/z*	*m/z*	*m/z*	*m/z*	*m/z*	*m/z*
		**I**_**424**_**/I**_**221**_		**I**_**610**_**/I**_**407**_		**I**_**626**_**/I**_**423**_	**I**_**829**_**/I**_**423**_
Pls_COL	221.08	424.16	407.17	610.23	423.14	626.25	829.30
		**2.7**		**3.9**		**3.0**	**17.1**
Pls_COL*sdgA/sdgB*	221.08	424.17	407.16	610.23	423.15	626.27	829.31
		**1.2**		**1.7**		**1.8**	**5.0**

### Functional characterization of the glycosylation of Pls

#### Pls reduces the adherence of *S*. *aureus* to Fg, Fn, or endothelial cells independently of its glycosylation status

**Fig 7:**
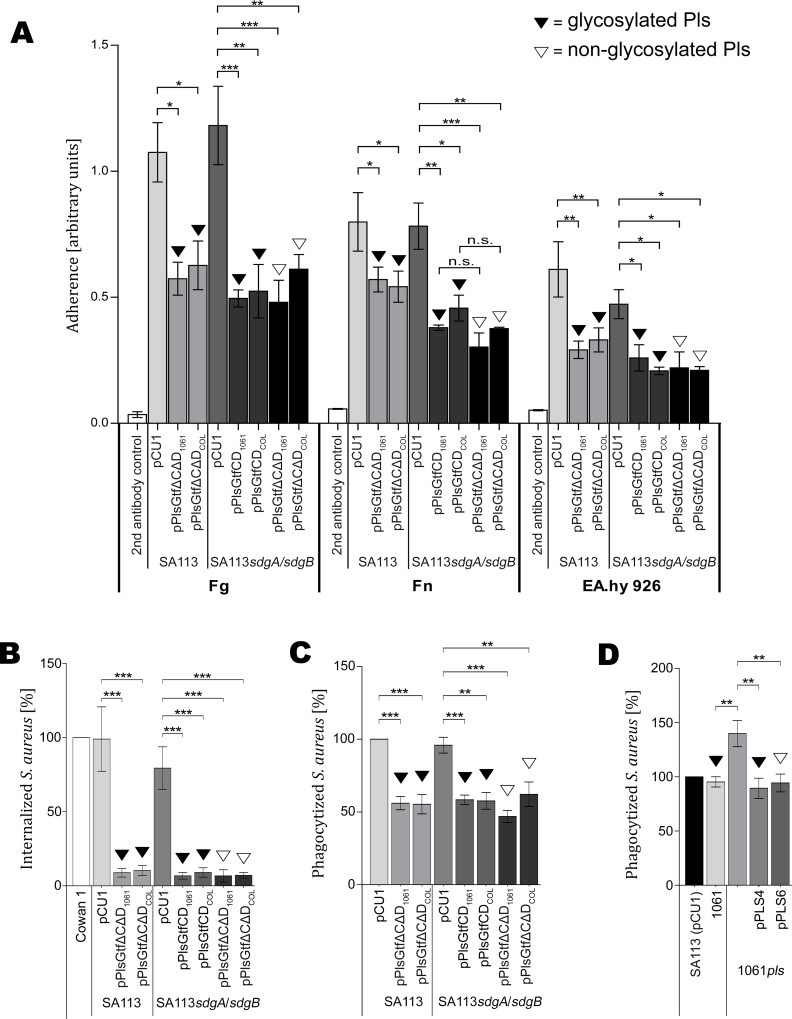
Functional characterization of Pls glycosylation. **(A)** Pls reduces the adherence of *S*. *aureus* to Fg, Fn, and endothelial cells independently of its glycosylation status. The wells of microtiter plates were coated with Fg, Fn or endothelial cells, blocked, and incubated with the bacteria. After washing, binding was assessed as arbitrary units in ELISA adherence assays. Results are shown as the mean of three independent experiments. Statistical significance is marked by asterisks. **(B)** Pls reduces the internalization of *S*. *aureus* by endothelial EA.hy 926 cells independently of its glycosylation status. The internalization of FITC-labeled *S*. *aureus* strains by adherent EA.hy 926 cells was assessed by flow cytometry and computed in relation to *S*. *aureus* strain Cowan 1, which was set to 100% internalization. Data are shown as the mean of three independent experiments. Statistical significance is marked by asterisks. **(C, D)** Pls reduces the phagocytosis of *S*. *aureus* by PMNs independently of its glycosylation status. The phagocytosis of FITC-labelled *S*. *aureus* strains by PMNs was assessed by flow cytometry and computed in relation to *S*. *aureus* SA113 (pCU1), which was set to 100%. Data are shown as the mean of three independent experiments. Statistical significance is marked by asterisks.

Previously, it has been reported that MRSA strains that naturally express *pls* as well as MSSA strains that recombinantly express *pls* revealed a markedly reduced adherence to the extracellular matrix and plasma proteins Fg, Fn, IgG, and laminin [29, 41, 42]. The mechanism of this function was proposed to be steric hindrance [29]. To test the possibility that modifying glycosyl residues contribute to that function by enhancing steric hindrance, we performed ELISA adherence assays and analyzed the adherence of SA113 strains recombinantly expressing *pls* from strain 1061 or from strain COL and producing either glycosylated or non-glycosylated Pls (Fig 7A). The control strains SA113 (pCU1) and SA113*sdgA/sdgB* (pCU1) containing the empty vector revealed pronounced and very similar adherence among each other to Fg or Fn. All strains producing Pls showed a significantly reduced adherence to Fg and Fn in comparison to their control strains. However, there was no significant difference in adherence to Fg or Fn among the strains producing a glycosylated version of Pls [SA113 (pPlsGtfΔCΔD_1061_), SA113 (pPlsGtfΔCΔD_COL_), SA113*sdgA/sdgB* (pPlsGtfCD_1061_) and SA113*sdgA/sdgB* (pPlsGtfCD _COL_)], versus a non-glycosylated version of Pls [SA113*sdgA/sdgB* (pPlsGtfΔCΔD_1061_) and SA113*sdgA/sdgB* (pPlsGtfΔCΔD_COL_)]. Moreover, there was no difference in the binding properties among the strains expressing *pls* from strain 1061 and from strain COL. Binding to the negative control (microtiter plate coated with blocking solution) was negligible. Furthermore, we analyzed the adherence of these strains to EA.hy 926 endothelial cells and obtained very similar results (Fig 7A). Thus, the glycosylation of Pls does not apparently contribute to the steric hindrance causing the diminished adherence to host structures.

### Pls reduces the internalization of *S*. *aureus* by human host cells independently of its glycosylation status

It has been reported before that MRSA strains that naturally express *pls* as well as MSSA strains that recombinantly express *pls* are internalized by non-professional phagocytes, such as host endothelial cells to a significantly lesser extent, which was also proposed to be due to steric hindrance [29, 42]. To analyze, whether the Pls-mediated prevention of internalization of *S*. *aureus* strains by human host cells is dependent on its glycosylation, we analyzed the strains described above producing glycosylated or non-glycosylated Pls for their internalization by EA.hy 926 endothelial cells using flow-cytometric internalization assays. The control strain SA113 (pCU1) was internalized by the endothelial cells at a similar rate like the strain Cowan 1 that is known to have a high capacity for internalization and was set to 100% internalization (Fig 7B). Similarly, the control strain SA113*sdgA/sdgB* (pCU1) was internalized by the endothelial cells at a high level, although its internalization rate seems slightly reduced compared to its parent. All strains producing Pls showed a significant and strong reduction of the internalization rate by endothelial cells in comparison to their control strains (Fig 7B). However, there was no significant difference in the internalization rate among the strains producing a glycosylated version of Pls [SA113 (pPlsGtfΔCΔD_1061_), SA113 (pPlsGtfΔCΔD_COL_), SA113*sdgA/sdgB* (pPlsGtfCD_1061_), SA113*sdgA/sdgB* (pPlsGtfCD_COL_)] versus a non-glycosylated version of Pls [SA113*sdgA/sdgB* (pPlsGtfΔCΔD_1061_), SA113*sdgA/sdgB* (pPlsGtfΔCΔD_COL_)] (Fig 7B). Moreover there was no significant difference in the internalization rate between the strains expressing the genes from strain 1061 or COL. Thus, the glycosylation of Pls does not seem to influence the internalization rate by human endothelial cells.

### Pls decreases the phagocytosis of *S*. *aureus* by polymorphonuclear neutrophils (PMNs) independently of its glycosylation status

To study the potential impact of Pls and its glycosylation status on the phagocytosis of *S*. *aureus* by professional phagocytes, we performed a flow-cytometric phagocytosis assay. The phagocytosis of the control strain SA113 (pCU1) by PMNs was set to 100% phagocytosis (Fig 7C and 7D). The control strain SA113*sdgA/sdgB* (pCU1) was phagocytosed by PMNs at a similar level (Fig 7C). All strains producing Pls showed a significant reduction of the phagocytosis rate in comparison to their control strains (Fig 7C). However, there was no significant difference in the phagocytosis rate among the strains producing a glycosylated version of Pls [SA113*sdgA/sdgB* (pPlsGtfCD_1061_), SA113*sdgA/sdgB* (pPlsGtfCD_COL_)] versus a non-glycosylated version of Pls [SA113*sdgA/sdgB* (pPlsGtfΔCΔD_1061_), SA113*sdgA/sdgB* (pPlsGtfΔCΔD_COL_)]. There was also no difference between the strains expressing the genes from strain 1061 or COL. Similarly, there was no significant difference in the phagocytosis rate between the strains 1061 and the 1061*pls* mutant expressing the different subclones of *pls* (Fig 7D). In contrast, the 1061*pls* mutant was phagocytosed at a significantly higher rate (Fig 7D).

### Biofilm formation of *S*. *aureus* Newman is enhanced upon expression of GtfC/GtfD-glycosylated Pls

**Fig 8:**
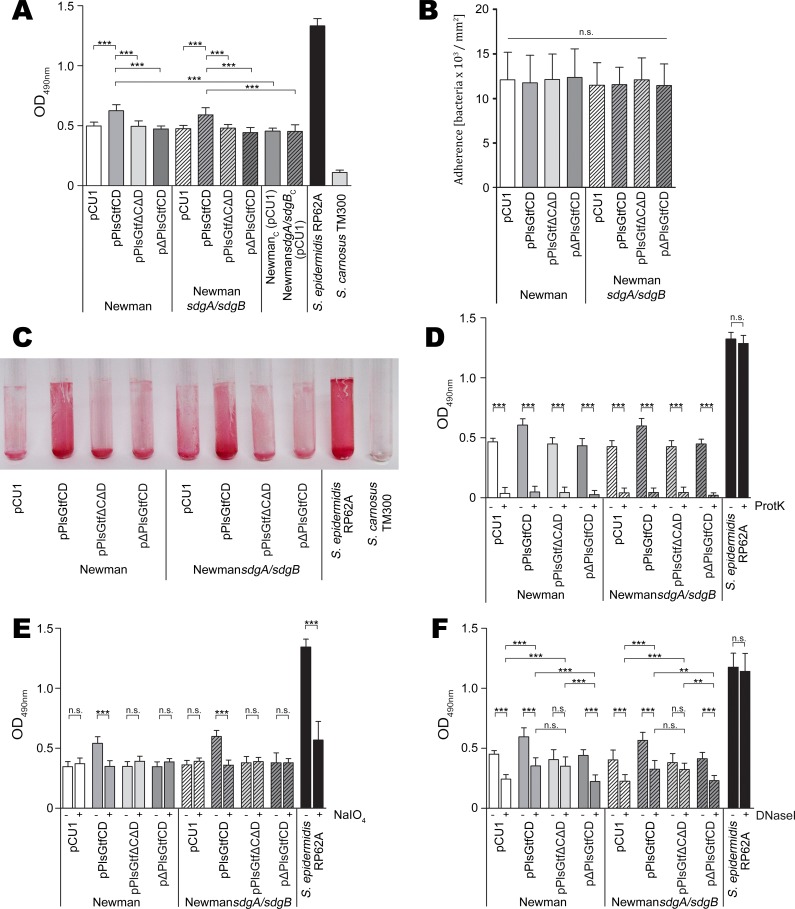
Expression of glycosylated Pls increases biofilm formation of *S*. *aureus* Newman. **(A)** Quantitative assay of biofilm formation. Strains were grown in TSB in microtiter plates. *S*. *epidermidis* RP62A and *S*. *carnosus* TM300 served as positive and negative controls, respectively. Data are shown as the mean of four independent experiments. Statistical significance is marked by asterisks. **(B)** Initial attachment to a plastic surface. Attached bacterial cells were analysed by phase-contrast microscopy, photographed and counted. Each assay was performed in triplicates. Data are shown as the mean of three independent experiments. **(C)** Biofilm formation on a glass surface. *S*. *epidermidis* RP62A and *S*. *carnosus* TM300 served as positive and negative controls, respectively. **(D)** Proteinase K (0.1 mg/ml) or **(E)** NaIO_4_ (40 mM) treatment (+) of preformed biofilms in microtiter plates and untreated controls (-). *S*. *epidermidis* RP62A served as a control. Data are shown as the mean of three independent experiments. Statistical significance is marked by asterisks. **(F)** Biofilm formation in the presence (+) or absence (-) of DNase I (0.1 mg/ml). *S*. *epidermidis* RP62A served as a control. Data are shown as the mean of three independent experiments. Statistical significance is marked by asterisks.

Pls has been reported to mediate cell-cell interaction [43]. To address the question, whether Pls mediates biofilm formation in a glycosylation-dependent manner, we analyzed the biofilm forming capacities of strains harboring the different *pls* and *gtfC/gtfD* expression plasmids in a polystyrene microtiter plate. Strains SA113 and SA113*sdgA/sdgB* expressing *pls* did not show increased biofilm formation probably because these strains form a strong polysaccharide intercellular adhesin (PIA)-dependent biofilm [44] thereby masking other factors (S2 Fig). However, we found that strains Newman (pPlsGtfCD_COL_) and Newman*sdgA/sdgB* (pPlsGtfCD_COL_) producing Pls glycosylated by GtfC/GtfD formed significantly higher levels of biofilm (*P* ≤ 0.001) than their negative controls carrying the empty vector (Fig 8A). Wells of representative biofilms stained with safranin are shown in the supplemental S2 Fig. Moreover, they also formed significantly higher levels of biofilm (*P* ≤ 0.001) than the respective strains producing Pls and non-functional GtfC/GtfD [Newman (pPlsGtfΔCΔD_COL_) and Newman*sdgA/sdgB* (pPlsGtfΔCΔD_COL_)] (Fig 8A). To ensure that increased biofilm formation is indeed due to GtfC/GtfD-glycosylated Pls, we also constructed strains expressing functional *gtfC*/*gtfD*, but not *pls* [Newman (pΔPlsGtfCD_COL_) and Newman*sdgA/sdgB* (pΔPlsGtfCD_COL_)], which produced significantly lower levels of biofilm (*P* ≤ 0.001) than strains expressing the intact *pls* (Fig 8A). Thus, the effect of increased biofilm formation in strains Newman (pPlsGtfCD_COL_) and Newman*sdgA/sdgB* (pPlsGtfCD_COL_) clearly depends on Pls and its glycosylation by GtfC/GtfD.

Strain Newman is known to harbor a variation of the SaeRS regulatory locus [45–47]. A nucleotide exchange within the *saeS* gene results in an exchange from leucine at aa position 18 (present in other *S*. *aureus* strains, *saeS*^*L*^) to proline (*saeS*^*P*^) [45–47]. This aa exchange leads to a constitutively expressed SaeRS system in strain Newman that has multiple consequences, one of them being reduced biofilm formation [47]. To verify that the increase in biofilm formation in strains Newman (pPlsGtfCD_COL_) and Newman*sdgA/sdgB* (pPlsGtfCD_COL_) is due to the expression of GtfC/GtfD-glycosylated Pls and not due to a point mutation resulting in the *saeS*^*L*^ allele, which would also lead to increased biofilm formation, we sequenced the *saeRS* locus in both strains and found no sequence alteration in comparison to the published *S*. *aureus* Newman genome sequence. Thus, we can rule out that a mutation within the *saeRS* regulatory locus caused the phenotype. To further exclude that any other mutation in the genome of strains Newman (pPlsGtfCD_COL_) and Newman*sdgA*/*sdgB* (pPlsGtfCD_COL_) caused the observed phenotype of increased biofilm formation, we cured both strains from their plasmids generating strains Newman_cured(C)_ and Newman*sdgA*/*sdgB*_C_. To enable equal growth conditions among all strains in the biofilm assay (i.e. supplementation with antibiotics), we transformed the cured strains with the empty vector generating strains Newman_C_ (pCU1) and Newman*sdgA*/*sdgB*_C_ (pCU1). In the biofilm assays, strains Newman_C_ (pCU1) and Newman*sdgA*/*sdgB*_C_ (pCU1) showed significantly lower biofilm formation (*P* ≤ 0.001) than strains Newman (pPlsGtfCD_COL_) and Newman*sdgA*/*sdgB* (pPlsGtfCD_COL_) and similar biofilm forming capacities like strains Newman (pCU1) and Newman*sdgA*/*sdgB* (pCU1) (Fig 8A) further verifying that indeed the production of GtfC/GtfD-glycosylated Pls is the cause of increased biofilm formation.

Generally, biofilm formation proceeds in two steps: Rapid initial attachment of the bacteria to a surface is followed by a more prolonged accumulation phase, which requires intercellular adherence [17]. Intercellular adherence may be mediated by protein factors or PIA, a polysaccharide whose production is encoded by the *icaADBC* operon [17]. Another important structural component of *S*. *aureus* biofilms is extracellular (e)DNA [48]. To characterize the mechanisms involved in the increased biofilm formation mediated by GtfC/GtfD-glycosylated Pls, we analyzed the initial attachment of the bacteria to a plastic surface. We could not detect any significant differences in the number of attached bacteria among the strains tested suggesting that increased intercellular adherence must account for the observed differences in biofilm formation (Fig 8B). In agreement, strains Newman (pPlsGtfCD_COL_) and Newman*sdgA/sdgB* (pPlsGtfCD_COL_) producing GtfC/GtfD-glycosylated Pls also showed increased biofilm formation on a glass surface (Fig 8C). To further dissect the mechanisms underlying the stimulated biofilm formation by GtfC/GtfD-glycosylated Pls, we treated preformed biofilms with proteinase K as well as with sodium metaperiodate (NaIO_4_). Proteinase K treatment completely abolished biofilm formation of all strains tested except for the control *S*. *epidermidis* RP62A, which is known to form a PIA-dependent biofilm, confirming the protein dependency not only of the biofilms mediated by GtfC/GtfD-glycosylated Pls, but also of strains Newman in general (Fig 8D). Interestingly, treatment with NaIO_4_, which oxidizes carbohydrates and is known to disintegrate PIA-dependent biofilms, significantly (*P* ≤ 0.001) degraded only the biofilms of strains Newman (pPlsGtfCD_COL_) and Newman*sdgA/sdgB* (pPlsGtfCD_COL_) producing GtfC/GtfD-glycosylated Pls to the levels of the remaining Newman strains strongly suggesting a direct involvement of the Pls sugar residues in biofilm formation (Fig 8E). As expected, biofilms of the PIA-producing control *S*. *epidermidis* RP62A were also significantly degraded (*P* ≤ 0.001) (Fig 8E). Furthermore, growth in the presence of DNase I significantly (*P* ≤ 0.001) reduced the biofilm levels of strains Newman (pCU1), Newman*sdgA/sdgB* (pCU1), Newman (pΔPlsGtfCD_COL_) and Newman*sdgA/sdgB* (pΔPlsGtfCD_COL_), which all did not produce Pls, suggesting that eDNA is an important structural component of strain Newman biofilms (Fig 8F). However, the biofilm levels of strains Newman (pPlsGtfΔCΔD_COL_) and Newman*sdgA/sdgB* (pPlsGtfΔCΔD_COL_) producing Pls not glycosylated by GtfC/GtfD were not noticeably altered when biofilms were grown in the presence of DNase I (Fig 8F). In contrast, the higher biofilm levels of strains Newman (pPlsGtfCD_COL_) and Newman*sdgA/sdgB* (pPlsGtfCD_COL_) producing GtfC/GtfD-glycosylated Pls were significantly (*P* ≤ 0.001) reduced when grown in the presence of DNase I to the levels of strains Newman (pPlsGtfΔCΔD_COL_) and Newman*sdgA/sdgB* (pPlsGtfΔCΔD_COL_) and still remained significantly (*P* ≤ 0.001 or *P* ≤ 0.01) higher than those of Newman strains not producing Pls when grown in the presence of DNase I (Fig 8F). These results together strongly suggest that two distinct mechanisms are involved in biofilm formation mediated by GtfC/GtfD-glycosylated Pls, one depending on GtfC/GtfD-glycosylated Pls and potentially also on eDNA, while the other being independent of glycosylation by GtfC/GtfD as well as of eDNA. While with strains Newman (pPlsGtfCD_COL_) and Newman*sdgA/sdgB* (pPlsGtfCD_COL_) both mechanisms can be observed, strains Newman (pPlsGtfΔCΔD_COL_) and Newman*sdgA/sdgB* (pPlsGtfΔCΔD_COL_) only display the latter.

## Discussion

In the past two decades, evidence has grown that bacterial glycoproteins play important roles in the physiology and pathophysiology of Gram-negative and Gram-positive bacteria, such as adherence to host cells, interaction with the host immune system, immune evasion, surface recognition, enzymatic activity, protein stability, and conformation [5–7, 10]. The knowledge on glycosylated surface proteins in *S*. *aureus*, the underlying glycosylation machinery and their potential role in pathogenesis has been very limited so far. In a search for staphylococcal surface glycoproteins, we identified four glycosylated surface proteins from the MRSA strain COL and two from strain 1061.

The ∼250- and ∼165-kDa glycoproteins from strain COL and the ∼175-kDa glycoprotein from strain 1061 were identified as the plasmin-sensitive protein Pls by MS. The presence of the *pls* gene usually is associated with the SCC*mec* type I, but has also been found in one strain harboring the SCC*mec* type IV [42]. Pls is sensitive to proteolysis by plasmin leading to 175-kDa and 68-kDa cleavage products [26]. However, these cleavage products also occur in lysostaphin lysates without prior proteolytic treatment [28]. Cleavage could be prevented (sometimes only partially) by the addition of protease inhibitors suggesting that Pls is cleaved by an *S*. *aureus* protease at the same position (between position R^387^ and A^388^ in Pls from strain 1061 [26]). This explains the presence of the large ∼250-kDa Pls glycoprotein in staphylococcal surface protein preparations or sometimes its absence due to proteolytic cleavage.

The expression of *pls* encoded on plasmid pPLS4 in the MSSA strains SA113, SH1000, and Newman led to the production of a glycosylated version of Pls, which however was not produced by the respective *sdgA/sdgB* mutant strains (Fig 2A and 2B) demonstrating that SdgA/SdgB are capable of transferring glycosyl residues to Pls. In contrast GtfA, which mediates the glycosylation of SraP [20, 21], is not apparently involved in the glycosylation of Pls as there was no glycosylated Pls detectable in the *sdgA/sdgB* mutants and the glycosylation of Pls seemed to be unchanged in the SA113*gtfA* mutant. However, the COL*sdgA/sdgB* mutant still produced a glycosylated version of Pls (Fig 2B) thereby demonstrating that the genome of strain COL must contain additional *gtf* genes that are able to confer glycosylation of Pls. In a search for potential *gtf* genes in strain COL, we identified *gtfC* and *gtfD* encoded downstream of the *pls* gene on the SCC*mec*. Expression and deletion analysis in the strains *S*. *aureus* SA113*sdgA/sdgB* and *S*. *carnosus* TM300 revealed that both *gtfC* and *gtfD* are involved in the glycosylation of Pls (Fig 3 and Fig 4).

Interestingly, we observed a difference among the genes cloned from strain COL in comparison with those cloned from strain 1061: While with strain COL, only *gtfD* is required for an initial glycosylation of Pls, with strain 1061, both *gtfC* and *gtfD* are required. Nucleotide sequence analysis revealed that the deduced aa sequences of GtfC_COL_ and GtfC_1061_ are 100% identical and that there is only one aa exchange between GtfD_COL_ and GtfD_1061_ (F^208^ ⇒ S^208^) potentially accounting for the observed difference. However, another possibility could be that sequence differences between Pls from strain COL and Pls from strain 1061 are responsible for the observed difference.

Furthermore, it seems likely that in strain COL SdgA/SdgB additionally to GtfC/GtfD transfer carbohydrate residues to Pls. Indeed, our mass-spectrometric analysis indicate that SdgA/SdgB are also involved in the glycosylation of Pls, because Pls purified from the strain *S*. *aureus* COL is more heavily glycosylated than Pls purified from the strain COL*sdgA/sdgB* (Table 2). Our mass-spectrometric analysis demonstrated that modifying carbohydrates are *N*-acetylhexosaminyl residues. Future analysis of the modifying glycan moieties of Pls prepared from different *gtf* mutants will clarify, whether further Gtfs might be involved in the glycosylation of Pls and whether the modifying sugars consist of one or more than one species of *N*-acetylhexosamines. In analogy to GspB and other SRR surface proteins, we expect *N*-acetylglucosaminyl and/or *N*-acetylgalactosaminyl residues to be among the modifying carbohydrates [12, 49, 50].

Equivalent to other reported SRR proteins (see below), we hypothesize a role for the glycan modifications in the function of Pls. As it was reported for Pls to promote bacterial cell aggregation [43], a possible function of the Pls glycosyl residues is an involvement in Pls-mediated cell aggregation and biofilm formation. Indeed, we could identify a role for the Pls glycosyl residues in biofilm formation in strain Newman (Fig 8). Analysis of the participating factors in biofilm formation mediated by GtfC/GtfD-glycosylated Pls revealed a proteinase K-sensitive factor as expected, which is also in agreement with Hazenbos et al. (2013), who found that proteinase K treatment of protein preparations resulted in a loss of reactivity with a monoclonal antibody that exclusively detected SD-repeat protein domains when they are glycosylated [15]. Most importantly, our further results strongly suggest that Pls mediates biofilm accumulation via two distinct mechanisms. The first mechanism requires Pls SD-repeat glycosylation by GtfC/GtfD and its sensitivity to NaIO_4_ indicates a direct involvement of the carbohydrate modifications in intercellular adherence. To our knowledge this is the first study to demonstrate the importance of sugar modifications of a staphylococcal surface glycoprotein in biofilm formation. This mechanism may involve the contribution of eDNA, because we observed a significant reduction of biofilm levels of strains Newman (pPlsGtfCD_COL_) and Newman*sdgA/sdgB* (pPlsGtfCD_COL_) by DNase I. However, we cannot exclude the possibility that this observation may at least partially be due to an overlap with the intrinsic eDNA dependency of strain Newman. The second mechanism of Pls-mediated biofilm formation seems to be independent of glycosylation as well as eDNA and becomes only evident upon biofilm growth of the strains Newman (pPlsGtfCD_COL_), Newman*sdgA/sdgB* (pPlsGtfCD_COL_), Newman (pPlsGtfΔCΔD_COL_), and Newman*sdgA/sdgB* (pPlsGtfΔCΔD_COL_) in the presence of DNase I suggesting that otherwise this second mechanism is masked by the presence of eDNA. The second, glycosylation-independent mechanism likely involves the Pls G5 domains (see Fig 3). G5 domains are also part of the Pls-homologous proteins Aap from *S*. *epidermidis* and SasG from *S*. *aureus* and known to promote biofilm formation via a zinc-dependent self-association mechanism [51–55]. Aap and SasG however lack an SRR domain and therefore the G5 domain-mediated mechanism of biofilm formation must be independent of glycosylation. Taken together, the findings resulting from our cloning, expression, and biofilm studies enabled us to propose two distinct mechanisms involved in biofilm formation mediated by GtfC/GtfD-glycosylated Pls and it may be speculated that the bacteria depending on the actual environmental conditions may apply one or the other. Further studies are planned in the future to exactly decipher the mechanisms underlying biofilm accumulation mediated by GtfC/GtfD-glycosylated Pls.

Strain Newman carries a variant *saeS*^*P*^ allele instead of the *saeS*^*L*^ allele resulting in an over-active SaeRS regulatory system [46, 47, 56]. Our findings suggest that the effect of increased biofilm formation mediated by GtfC/GtfD-glycosylated Pls depends on the *saeS*^*P*^ allele. Although generally, the *saeRS* system seems to be quite conserved, it was recently reported that the *saeS*^*P*^ allele is present in several uncharacterized *S*. *aureus* strains found at the Genomes OnLine Database (GOLD) (https://gold.jgi.doe.gov) (IDs 53133–53147) [47]. Further analyses are required to determine, whether the *saeS*^*P*^ allele might also be an occasional or even frequent feature of clinical MRSA. Similarly, variations can also occur in other regulatory loci: a substantial number of clinical *S*. *aureus* isolates have been found to be negative in the well-characterized accessory gene regulator *agr* [57]. Alternatively, it seems possible that an upregulated *saeRS* system via the *saeS*^*P*^ allele may not be required in the *in vivo* situation. In support of this, several analyses revealed that *saeRS* is an *in vivo*-active and essential regulatory locus that plays a crucial role in *S*. *aureus* virulence, which has also been shown during human and mouse infection with MRSA [58–61]. However, the regulatory mechanisms underlying increased biofilm formation mediated by GtfC/GtfD-glycosylated Pls in strain Newman still have to be elucidated.

It has been previously established that Pls decreases the *S*. *aureus* adherence to extracellular matrix and plasma proteins including Fg, Fn, IgG, and laminin and also its internalization by human host cells by the mechanism of steric hindrance [29, 41, 42]. To study whether the glycosyl residues of Pls mediate steric hindrance, we performed different functional ELISA and flow-cytometric assays. We could neither detect an impact of the sugar modification on the Pls-mediated decrease of *S*. *aureus* SA113 adherence to Fg, Fn, and endothelial cells, nor in the decrease of its internalization by endothelial cells or of its phagocytosis by PMNs. Thus, we can rule out the possibility that the steric hindrance is caused by the glycosyl residues masking other surface adhesins and leading to the observed effects of Pls.

The >300 kDa glycosylated surface protein produced by strains COL and 1061 (Fig 1) probably represents SraP (SasA) [20], because we identified a glycosylated protein with the same size produced by the strain SA113*sdgA*/*sdgB* as SraP by MS (S1 Fig). The ∼120-kDa glycoprotein identified from strain COL that was missing from the COL*sdgA/sdgB* mutant (Fig 2B) might either be SdrC, SdrD, or SdrE [62], because *sdgA/sdgB* are located downstream of the *sdrCDE* locus and the genes encoding Gtfs are frequently encoded adjacent to the structural genes, whose products they glycosylate [12, 49, 50]. Alternatively, the ∼120-kDa glycoprotein might be ClfA [15, 16]. The absence of the ∼120-kDa glycoprotein from strain 1061 might be explained by non-functional *sdgA/sdgB* genes in strain 1061. Interestingly, our nucleotide sequence analysis revealed the insertion of an IS*1181* element upstream of the *sdgB* gene in strain 1061 thereby potentially influencing *sdgB* transcription (S3 Fig). Alternatively or additionally, a non-functional SdgA or SdgB might be explained by 6 and 3 aa exchanges found in SdgA and SdgB from strain 1061, respectively, compared to SdgA and SdgB from strain COL (which are identical to SdgA and SdgB from strain SA113).

Besides SraP and ClfA from *S*. *aureus* [14–16] and GspB from *S*. *gordonii* [63], further members of the growing family of SRR surface proteins include Hsa from *S*. *gordonii*, which is homologous to GspB [64], SrpA of *Streptococcus sanguis* [50], PsrP of *Streptococcus pneumoniae* [24], Srr1 and its homolog Srr2 from *Streptococcus agalactiae* [65], and Fap1 from *Streptococcus parasanguinis* [11, 12]. SRR proteins have been associated with different adhesive functions and with bacterial pathogenesis. Like SraP and GspB, SrpA binds to platelets and it has been shown in animal models of infective endocarditis that their expression is associated with a higher pathogenicity [14, 50, 66]. With GspB, it has been demonstrated that incorrect glycosylation leads to impaired binding to its platelet receptor [49]. Srr1 mediates binding to several types of human epithelial cell lines and interacts with cytokeratin 4 as an epithelial cell surface ligand, which seems to involve the glycosylated SRR domain of Srr1 [67]. Furthermore, it was shown that the extent of Srr1 glycosylation by GtfCDEFGH modulates the adherence and virulence of *S*. *agalactiae* in a rat model of neonatal sepsis [65]. The fimbria-associated protein Fap1 from *S*. *parasanguinis* that colonizes saliva-coated teeth thereby causing the formation of dental plaque mediates biofilm formation in an *in vitro* tooth model, which seems to involve the sugar residues [68, 69]. PsrP from *S*. *pneumoniae* binds to keratin 10 on lung epithelial cells and mediates bacterial cell aggregation [70, 71]. Similarly, it was shown that GspB and SraP promote bacterial aggregation [71]. Thus, in several SRR proteins, the glycan moieties of the proteins seem to be involved in or to modulate the functions of the respective adhesins, which is in line with our finding that Pls confers increased biofilm formation when glycosylated by GtfC/GtfD.

Although there are several common features among the SRR proteins of Gram-positive cocci and the *pls* locus shares some of them, such as the structural gene encoding a large SRR surface protein and *gtf* genes that are located downstream of the structural gene and encode enzymes involved in posttranslational modification, there also seem to be marked differences. Like GtfA from the *S*. *aureus sraP* locus and GtfA from the *S*. *gordonii* M99 *gspB* locus, which share more than 40% identical aa with the poly (glycerol-phosphate) α-glucosyltransferase TagE of *Bacillus subtilis* [13], GtfC also has a high degree of identical aa with poly (glycerol-phosphate) α-glucosyltransferases (see above) suggesting similar functions of GtfA and GtfC. Here, we found that *S*. *carnosus* and the SA113*sdgA/sdgB* mutant produced non-glycosylated, surface-anchored Pls upon *pls* expression, when *gtfC*/*gtfD* are either not present or deleted. In contrast, in a *gtfA* and *orf4* (later termed *gtfB*) mutant of *S*. *gordonii*, GspB was not detectable [19]. This was not due to an altered *gspB* transcription in these mutants. Thus, the authors concluded that either GspB is not translated or quickly degraded intracellularly and thus the Gtfs may greatly affect the stability of GspB [13, 19]. Similarly, GtfA and/or GtfB is essential for the production of Srr1, while full glycosylation of Srr1 mediated by the six dispensable additional Gtfs (GtfCDEFGH) leads to the cell surface display of a protein that is protected from proteolysis [65]. Moreover, the non-glycosylated Fap1 protein is less stable and more sensitive to protein degradation upon inactivation of the *gtf* gene that mediates glycosylation of Fap1 [69]. Recently, modifying glycosyl residues have also been demonstrated to protect ClfA from proteolytic cleavage by host proteases and might therefore modulate its function as an adhesin [15]. However, in our preliminary experiments, we could not detect a difference in protein stability or secretion of glycosylated versus non-glycosylated Pls (Fig 3).

In conclusion, Pls is a glycoprotein and GtfC/GtfD as well as SdgA/SdgB are involved in its glycosylation. The production of GtfC/GtfD-glycosylated Pls leads to increased biofilm, while glycosyl residues do not have an impact on other previously known Pls properties. Because Pls has been shown to be a virulence determinant in a mouse septic arthritis model [25], it is reasonable to assume that glycosyl residues might contribute to *in vivo* biofilm formation. Future experiments are planned to clarify, if the sugar modifications of Pls may represent promising new targets for therapeutic or prophylactic measures.

## Materials and Methods

### Bacterial strains, growth conditions, plasmids, phage, cell culture, and reagents

Bacterial strains used in this study are listed in Table 1. *Staphylococcus* and *Escherichia coli* strains were grown aerobically at 37°C in Tryptic Soy (TS) broth (TSB, BD Bioscience) and lysogeny broth (LB, BD Bioscience), respectively. TS and LB agar plates contained 1.4% agar. Staphylococcal cultures were cultivated in TSB unless otherwise indicated. Antibiotics were added, when appropriate: Ampicillin (Am; 100 μg/ml), chloramphenicol (Cm; 10 μg/ml), erythromycin (Em; 10 μg/ml), tetracycline (Tc; 10 μg/ml), and kanamycin (Kan; 25 μg/ml). For the cloning of *pls*, *gtfC*, and *gtfD*, the vector pCU1 was used [72]. The *sdgA/sdgB*-deficient *S*. *aureus* SA113 mutant was constructed by using the plasmids pEC2 and pBT2 [73]. The *sdgA/sdgB*-deficient *S*. *aureus* SH1000 mutant was constructed by using the plasmids pGL433 [74] and pMUTIN4 [75]. For the construction of the mutants SA113*gtfA* [20], SA113*gtfA*/*sdgA/sdgB*, and SA113*bgt*, the vector pKOR1 was employed [76]. Pls subclones were constructed from the plasmid pPLS4 [26]. For the transduction of the *sdgA/sdgB* double mutation from *S*. *aureus* SH1000*sdgA/sdgB* into strains COL and Newman, the phage Φ11 was used [77]. For the internalization and adherence assays, the endothelial cell line EA.hy 926 (ATCC CRL-2922) was employed [78]. Cultivation of the EA.hy 926 cells was performed as described [79].

Micro Bio-Spin P6 Columns were purchased from BIO-RAD (Munich, Germany). Trypsin, chymotrypsin, endoproteinase Glu-C, and pronase were from Roche Diagnostics GmbH (Mannheim, Germany). Thermolysin was purchased from Sigma-Aldrich Chemie GmbH (Taufkirchen, Germany). Methanol, formic acid, and acetic acid were from Fluka (Buchs, Switzerland). All solvents used were of HPLC grade purity.

### DNA manipulations, transformation, PCR, DNA sequencing, websites, and accession numbers

DNA manipulations and transformation of *E*. *coli* were performed according to standard procedures [80]. *S*. *carnosus* and *S*. *aureus* strains were transformed by protoplast transformation [81] or electroporation [82]. Plasmid DNA was isolated using the PrepEase MiniSpin Plasmid Kit (USB, Staufen, Germany) and staphylococcal chromosomal DNA was isolated with the PrestoSpin D Bug DNA purification kit (Molzym, Bremen, Germany). PCR was carried out using the Phusion High-Fidelity DNA Polymerase (Finnzymes, Vantaa, Finland) according to the instructions of the manufacturers. The primers (Table 3) were synthesized by Eurofins MWG Operon (Ebersberg, Germany). DNA sequences were determined by Eurofins MWG Operon using the indicated primers (Table 3) and an ABI 3730XL DNA sequencer. The DNA and deduced protein sequences were analyzed using the program JustBio at http://www.justbio.com. The deduced Pls and GtfC/GtfD sequences were compared using the programs BLASTP [83] and FASTA [84] and the alignments were done using the program ClustalW at the European Bioinformatics Institute (EBI, Cambridge, UK). The signals obtained by MS were assigned to peptides of known proteins by using the MASCOT search engine and the SwissProt database at http://www.expasy.ch. The CAZy database used for the identification of putative Gtfs encoded by the *S*. *aureus* COL genome is available at www.cazy.org.

**Table 3:** Primers used in this study.

Primer name	Oligonucleotide sequence (5´→ 3´; restriction site underlined)
TE-P1-PstI^1^	AACTGCAGCCTAAAATGTAATTCATATTATCGCCTC
TE-P3-HindIII^1^	CCCAAGCTTGGTTTAACAACAACAGGTGTTATTAAAGATGC
TE-P1-XbaI^1^	GAGTTATACAACTCTAGAGAGGTATAATAAAAACGCGC
TE-P4-EcoRI^1^	GGACATATCTTAGAATTCTTAACGGAGGAAAAAAATGACTGAATTTGATTATCG
Rmgts4^1^	TATATGAATTCATACACCTGTTAAACCAATGAGTAC
Rmgts5^1^	TTATAGGTACCGGCATTGCTTTTCTGTTGATAC
Rmgts6^1^	TTTAAGGTACCTACTGTGAAGCACAGCTACTAC
Rmgts7^1^	TATTAGGATCCGTACTTGTTGACCAGTATCAAAC
Fkan1^1^	GGCGGGGTACCCAGCGAACCATTTGAGG
Rkan2^1^	GGGGCGGTACCAATTCCTCGTAGGCGCTCGG
PlsGtfCD-F^1^	ATATGGTACCGGTATAGGGGGAGCCATTGT
PlsGtfCD-R^1^	ATATGGTACCGGATGATGCTTTAAAGAGTGTCG
Pls4Sub1-R^1^	GTCTGCATCAGAATCGCTATCTGCGTCTGAATCGCTGTCCGC
Pls4Sub2-R^1^	GCTGTCTGAATCGCTGTCCGCATCAGAGTCGCTATCTGCGTC
Pls4Sub3-R^1^	TGCGTCTGAGTCGCTGTCTGCATCAGAATCGCTATCTGC
Pls4Sub1/2/3-F^1^	AGAGATCATAATGACAAAACAGATAAACCAAATAATAAAGAG
GtfC-F^2^	GTAGTACTAATTTCTTACAAAATATG
GtfC-R^2^	CAATAAGTGAGTTGTCTCATATG
GtfD-F^2^	CAGGAGAATAACGTGCAACGG
GtfD-R^2^	GATCTATATGATTCAAGAGGCT
GtfC-mutF^2^	TGGCAGACGTCGTAAAAACA
GtfC-mutR^2^	TCAAGCACTCTAAAGCTTTTTCAA
GtfD-mutF^2^	AAAAACCCATCAAGAATACTAGGAA
GtfD-mutR^2^	GAACGACAAAACTTCACTGTTGA
Pls6B-F^2^	CGCGGATCCTACCAATGAATATGGTTGTTACAAATAG
Pls6K-F-1581^2^	TATGGTACCGCAGATAGAGATCATAATGACAAAACAGATAAACC
Pls6B-R-1637^2^	ATAGGATCCTTATTTTTCTTCATTATTTTTGTTTTTACGACGTCTGCC
Sub-SD-R^2^	ATCATCTTTAGCACCATGGATGATTACTTCATC
Pls-F^2^	GGTAATGTTCAAACTATTGAAC
Stop-Pls-R^2^	TTATTTTTCTTCATTATTTTTGTTTTTA
SdgAB-B-F^2^	ATATGGATCCAACGGCTCAAATAACGCAACG
SdgAB-X-R^2^	ATATCCCGGGATCGACACGAGAAGGTCGTT
SdgA-F2^2^	TCGTCCTCATGAATTAGGAAATG
SdgB-R2^2^	AACGTCCTGATGAAAAACGTG
1061-SdgB-F^2^	CAAATTGGTCCCATTGTTTAAT
1061-IS*1181*-R^2^	TGAACGATATGGAATCTGTCAAA
SaeS-F^2^	CCGTATTAGAGAAAAATTAGAAAAAGAGAGC
SaeS-R^2^	CAAAAAAAGAAGCCCTCATTAATGGG
SaeR-F^2^	GAGTCACTCATTGTTAAAACAGATTTCAC
SaeR-R^2^	ATGCAATTGCTAAAATAGTTGAAGTTAATGG

Primers used for ^1^cloning and ^2^sequencing.

The accession numbers of the deduced sequences of the UniProt and GenBank databases are: Pls (SACOL0050): Q5HJU7, AAW38699; GtfC (SACOL0051): Q5HJU6, AAW38700; GtfD (SACOL0052): Q5HJU5, AAW38701; SdgA (SACOL0611): Q5HIB1, AAW37720; SdgB (SACOL0612): Q5HIB0, AAW37721; poly (glycerol-phosphate) α-glucosyltransferase from *S*. *aureus* C75S: ACZ59060; poly (glycerol-phosphate) α-glucosyltransferase from *S*. *epider-midis* ATCC 12228: NP_765949. The nucleotide sequence accession numbers are for the *sdgA/sdgB* genes from strain *S*. *aureus* 8325–4: SAOUHSC_00547 (*sdgA*) and SAOUHSC_00548 (*sdgB*), for *gtfA*: SAOUHSC_02984 [20], and for the putative bactoprenol glycosyltransferase *bgt*: SAOUHSC_00713. The GenBank nucleotide sequence accession number for the *gtfC*/*gtfD* genes from strain *S*. *aureus* 1061 is JX193902 and for the *sdgA/sdgB* genes including the adjacent sequence of the insertion sequence IS*1181* from strain *S*. *aureus* 1061 is JX204384. The Pfam accession number for the G5 domain is available at http://pfam.xfam.org/family/PF07501.

### Construction of the SA113*sdgA*/*sdgB*, SA113*gtfA*, SA113*gtfA*/*sdgA*/*sdgB*, SA113*bgt*, SH1000*sdgA*/*sdgB*, Newman*sdgA*/*sdgB*, and COL*sdgA*/*sdgB* mutant strains by gene replacement and phage transduction

The *sdgA/sdgB* genes are colocalized in the same locus on the chromosome. The double mutant *S*. *aureus* SA113*sdgA/sdgB* was created by the replacement of the *sdgA/sdgB* genes with the antibiotic resistance marker *ermB*. Briefly, both DNA fragments of approximately 1 kbp flanking the *sdgA/sdgB* locus were PCR amplified with the primer pairs TE-P3-HindIII/TE-P1-PstI and TE-P1-XbaI/TE-P4-EcoRI, respectively (Table 3). Both, the upstream and downstream DNA fragments were restricted, purified, and ligated into the pBT2 vector together with a 1.1 kbp PstI-XbaI fragment encoding the *ermB* gene taken from the plasmid pEC2. *S*. *aureus* SA113 was transformed with the resulting knock-out plasmid pBT-*sdgA/sdgB* by electroporation. By incubation at 42°C and subsequent screening for Em-resistant clones without the plasmid-encoded Cm resistance, the *sdgA/sdgB* mutant, was identified. Similarly, the mutants SA113*bgt*, SA113*gtfA*, and SA113*gtfA*/*sdgA/sdgB* were constructed by using the vector pKOR1. The *S*. *aureus* SH1000*sdgA/sdgB* mutant was constructed using the primer pairs Rmgts4/Rmgts5 and Rmgts6/Rmgts7 and the Kan resistance cassette, which was PCR amplified from the plasmid pGL433 using the primer pair Fkan1/Rkan2 (Table 3). To introduce the *sdgA/sdgB* mutation from strain SH1000*sdgA/sdgB* into strains COL and Newman, phage transduction was performed using Φ11 as the transducing phage as described [77].

### Construction of *pls* and *gtfC*/*gtfD* expression clones, corresponding *pls*, *gtfC* and/or *gtfD* deletion mutants, and *pls* subclones

To analyze the potential of GtfC and/or GtfD to glycosylate Pls, the *pls* gene and the downstream located genes, *gtfC* and *gtfD*, including the ribosomal binding sites and putative promoter sequences were amplified by PCR from *S*. *aureus* COL and 1061 genomic DNA using the primers PlsGtfCD-F and PlsGtfCD-R (Table 3) yielding a 9.81 kbp DNA and a 10.08 kbp fragment, respectively. The DNA fragments were cloned into the KpnI site of the shuttle vector pCU1 in *E*. *coli*, generating the plasmids pPlsGtfCD_COL_ and pPlsGtfCD_1061_. To functionally delete *gtfC* on the plasmids pPlsGtfCD_COL_ and pPlsGtfCD_1061_, the plasmid DNA was restricted by Eco47III and EcoRV and religated leading to a deletion of 478 bp and creating plasmids pPlsGtfΔCD_COL_ and pPlsGtfΔCD_1061_. To functionally delete *gtfD* on the plasmids pPlsGtfCD_COL_ and pPlsGtfCD_1061_, they were restricted by BglII. Then, the sticky ends were refilled by the Klenow fragment and religated generating a frameshift mutation and plasmids pPlsGtfCΔD_COL_ and pPlsGtfCΔD_1061_. Plasmids pPlsGtfΔCΔD_COL_ and pPlsGtfΔCΔD_1061_ were constructed by introducing the frameshift mutation in *gtfD* in the plasmids pPlsGtfΔCD_COL_ and pPlsGtfΔCD_1061_ as described above. To functionally delete *pls*, plasmid pPlsGtfCD_COL_ was restricted by HpaI and XbaI resulting in a deletion of 4,736 bp, the XbaI sticky end was made blunt end by the Klenow fragment and the DNA fragment was religated yielding plasmid pΔPlsGtfCD_COL_.

The sequences of the *pls* and *gtfC*/*gtfD* genes and their deletion derivatives were verified by DNA sequencing of the respective plasmids using the primers listed in Table 3. Subsequently, plasmids pPlsGtfΔCΔD_COL_ and pPlsGtfΔCΔD_1061_ were introduced into strain *S*. *aureus* SA113, all plasmids except for pΔPlsGtfCD_COL_ were introduced into *S*. *aureus* SA113*sdgA/sdgB* and *S*. *carnosus* TM300, and plasmid pΔPlsGtfCD_COL_ was introduced into strain *S*. *aureus* Newman and *S*. *aureus* Newman*sdgA/sdgB*.

To analyze the involvement of the SD-repeat region in the glycosylation of Pls, we constructed different subclones from plasmid pPLS4 [26] by using inverse PCR and the primer pairs: Pls4Sub1-R/Pls4Sub1/2/3-F to generate pPLSsub1 (9.51 kbp fragment), Pls4Sub2-R/Pls4Sub1/2/3-F to generate pPLSsub2 (9.56 kbp fragment), and Pls4Sub3-R/Pls4Sub1/2/3-F to generate pPLSsub3 (9.85 kbp fragment) (Table 3) (Fig 5A) in *E*. *coli*. After passaging the plasmids in *S*. *aureus* SA113, they were introduced into *S*. *aureus* 1061*pls*.

### Protein isolation, purification of Pls via lectins, SDS-PAGE, and periodic acid-Schiff’s (PAS) staining

Surface-associated proteins of staphylococcal strains were solubilized from the cell surface by heating with SDS-sample buffer essentially as described before [85]. Staphylococcal surface proteins covalently linked to the peptidoglycan were prepared by lysostaphin treatment of cultures that were grown overnight in TSB as described [86].

To prepare the lysostaphin lysates for the purification of Pls via lectins, staphylococcal strains were grown overnight in Todd-Hewitt broth (BD Bioscience). Cells were harvested, washed in phosphate-buffered saline (PBS), and resuspended in 40 ml PBS. Then, 200 μl of lysostaphin (5 μg/ml), 50 μl of DNase (1 mg/ml) and protease inhibitors (complete EDTA-free protease inhibitor cocktail; Roche) were added and incubated at 37°C for 2 h. The lysates were centrifuged at 13,000 rpm for 20 min. The supernatant was heated to 80°C to stop the reaction, centrifuged again, and sterile filtered. A 1 ml column packed with ConA sepharose 4B (GE Healthcare, München, Germany) was equilibrated with binding buffer (20 mM Tris-HCl, 0.5 M NaCl, pH 7.4) according to the instructions of the supplier. Afterwards, the lysate was applied to the column, the flow-through was collected and reapplied to the column thrice. The column was then washed with 25 ml binding buffer and bound protein was eluted with 10 ml elution buffer (binding buffer containing 15% methyl α-D-glucopyranoside) (Sigma Aldrich, München, Germany). The eluted fractions were separated by SDS-PAGE to check for the presence of protein. Fractions containing protein were passed through a NAP-10 G25 column (GE healthcare) to remove the small methyl α-D-glucopyranoside.

Staphylococcal surface, surface-associated or purified proteins were separated by SDS-PAGE (10% or 7.5% separation gel, 4.5% stacking gel) and stained with Coomassie Brilliant Blue G-250. Glycoproteins were detected using Pierce Glycoprotein Staining Kit (Thermo Scientific, Schwerte, Germany) in accordance with the protocol of the supplier by staining the sugar moieties directly in the SDS gel.

### Acid hydrolysis

50 μl of a Pls solution (5 to 15 pmol/μl, 50 mM Tris/HCL or 100 mM NH_4_HCO_3_, pH 7.4 to 7.8) were transferred to distilled water by use of Micro Bio-Spin P6 columns according to the manufacturer’s instructions. Briefly, the column was equilibrated with distilled water, the sample was applied to the column, and the protein was eluted with distilled water. 25 μl aliquots of the eluate were adjusted to 12.5% acetic acid in a total volume of 50 μl and incubated for 2 h at 95°C. Subsequently, the solvent was evaporated *in vacuo* and the residue was redissolved in 40% methanol/0.5% formic acid for mass-spectrometric analysis.

### Proteolytic cleavage

50 μl of a Pls solution (5 to 15 pmol/μl, 50 mM Tris/HCL or 100 mM NH_4_HCO_3_, pH 7.4 to 7.8) were rebuffered to 25 mM NH_4_HCO_3_ by use of Micro Bio-Spin P6 columns as described above. Aliquots corresponding to 100 to 200 pmol were incubated in the presence of trypsin, chymotrypsin, endoproteinase Glu-C (0.2 μg each) or pronase (1 μg) overnight at 37°C. For digests with thermolysin, 0.5 μg of the protease were added and the mixture was incubated overnight at 65°C.

### Mass spectrometry (MS)

The bands containing the glycosylated proteins were excised from the polyacrylamide gel and prepared for MS. For this, proteins were digested tryptically in the gel and the peptides were extracted, desalted, and subjected to electrospray ionization on a Q-Tof Premier coupled to a Nano Acquity (Waters Micromass, Eschborn, Germany) at the Integrated Functional Genomics (IFG) Core Unit of the Interdisciplinary Center of Clinical Research (IZKF) at the University Hospital of Münster (Germany). The obtained signals were assigned to peptides of known proteins by using the MASCOT search engine and the SwissProt database and the ProteinLynx Global SERVER (PLGS) software (Waters Micromass).

The products of acid hydrolysis and proteolytic cleavage were analyzed by nanoESI Q-Tof MS and MS/MS and chosen (glyco)peptide structures were deduced from fragment ion spectra derived from CID. NanoESI MS experiments were carried out by use of a quadrupole time-of-flight (Q-Tof) mass spectrometer (Micromass, Manchester, UK) equipped with a Z-spray source in the positive ion mode. The source temperature was kept at 80°C and the desolvation gas (N_2_) flow rate at 75 l per h. The capillary and cone voltages were adjusted to 1.1 kV and 30 V, respectively. For low energy CID experiments, the (glyco)peptide precursor ions were selected in the quadrupole analyzer and fragmented in the collision cell using a collision gas (Ar) pressure of 3.0 × 10^−3^ Pa and collision energies of 30–60 eV (*E*_lab_).

### ELISA adherence assay

The wells of 96-well microplates were coated with fibrinogen (Fg, 20 μg/ml; Calbiochem), fibronectin (Fn, 10 μg/ml; Roche), or as a negative control with blocking buffer (protein-free blocking buffer, Thermo Fisher Scientific) at 4°C overnight and subsequently blocked. To assess the adherence to endothelial cells, EA.hy 926 cells were grown to confluence in 96-well cell culture plates (Greiner Bio-One), washed with PBS, fixed with ice-cold methanol (Merck) and blocked with blocking buffer. Then, the microplates were washed thrice and each well was incubated for 2 h at 37°C with 100 μl of a staphylococcal suspension, which was previously grown overnight, washed with PBS, sonicated using an ultrasonic cell disruptor (Branson Sonifier 250) to separate cell aggregates, and adjusted to an optical density (OD_578_) of 1.0 (corresponding to approximately 5 x 10^8^ cfu/ml). As negative controls, wells without bacteria were included. Unbound bacterial cells were removed by washing twice with 200 μl PBS. Bound *S*. *aureus* cells were detected by a polyclonal rabbit anti-*S*. *aureus* antibody (previously raised in rabbits by Eurogentec, Liège, Belgium) (diluted 1:1,500) and alkaline phosphatase-conjugated goat anti-rabbit IgG (diluted 1:2,000 [0.32 μg/ml], Dako). SigmaFast *p*-Nitrophenylphosphate (Sigma Aldrich) conversion was detected by determination of the OD_405_ after 30 min of incubation.

### Preparation of FITC-labeled staphylococci and flow-cytometric internalization and phagocytosis assays

Overnight-grown staphylococci were washed, sonicated, fixed, and fluorescein isothiocyanate (FITC isomer I; Invitrogen)-labeled as described before [79]. Sample preparation and detection of internalized staphylococci by EA.hy 926 cells were performed by flow-cytometric internalization assays as described before [79].

The phagocytosis assay was performed as described [87]. Briefly, PMNs were freshly isolated from Na citrate-treated blood from healthy donors by density gradient centrifugation using Ficoll-Paque Plus (Amersham Bioscience) according to the manufacturer's instruction. FITC-labeled bacteria were added to the PMNs and incubated. Samples were analyzed on a FacsCALIBUR (BD Bioscience). Electronic gating was used to analyze 5,000 PMNs in each sample. The FL1 photomultiplier (transmittance at 500 nm) was used to detect uptake of staphylococcal cells by PMNs.

### Quantitative biofilm assay and initial attachment assay

For quantification of the biofilm-forming capacity, a biofilm assay was performed essentially as described previously [88]. Briefly, strains were grown in TSB for 24 h at 37°C in 96-wells polystyrene microtiter plates (cell star; Greiner, Frickenhausen, Germany). Afterwards, the plates were emptied, the wells were washed with PBS and adherent biofilms were stained with 0.1% safranin (Serva). In some experiments, 24-h biofilms were washed with PBS and then treated with 0.1 mg/ml proteinase K (Sigma) in 20 mM Tris-HCl (pH 7.5) or with 40 mM NaIO_4_ (Applichem) in double-distilled H_2_O for 2 h at 37°C. In the respective untreated controls, 24-h biofilms were incubated with 20 mM Tris-HCl (pH 7.5) or double-distilled H_2_O for 2 h at 37°C. Furthermore, in some experiments 24-h biofilms were grown in the presence of 0.1 mg/ml DNase I as described [89]. Afterwards, the wells were emptied, washed with PBS and stained with 0.1% safranin. Absorbance was measured with a Micro-ELISA-Autoreader at 490 nm. Strains were tested at least in quadruplicates. Determination of biofilm formation on a glass surface was carried out essentially in the same way, except that 5 ml TSB were inoculated in glass tubes. Initial attachment of the bacteria to a plastic surface was tested essentially as described before with some modifications [88]. Briefly, diluted bacterial cell suspensions in 2 ml PBS were incubated in the wells of a Nunc Lab-Tek Chamber Slide-System (Thermo Scientific) for 30 min at 37°C and after two washing steps, attached bacteria were evaluated by phase-contrast microscopy, photographed and counted; the number of adhered cells per square millimeter was determined.

### Ethics statement

All phagocytosis experiments were performed with the healthy adult blood donors giving written informed consent according to human experimentation guidelines. The study was conducted according to the principles expressed in the Declaration of Helsinki and was approved by the local ethics committee (Ethikkommission der Ärztekammer Westfalen-Lippe und der Medizinischen Fakultät der WWU Münster) (reference number: Sitzung 19.05.1999).

### Statistical analysis

Mean values of experimental data were compared with one-way ANOVA and, if adequate with subsequent Bonferroni’s posttest for multiple comparisons using GraphPad Prism 5. *P* values ≤ 0.05 were considered statistically significant and are indicated with asterisks: * (*P* ≤ 0.05), ** (*P* ≤ 0.01), and *** (*P* ≤ 0.001).

## Supporting Information

S1 Table Summary of (glyco)peptides derived from acid hydrolysis of Pls from *S. aureus* strain 1061 and detected by nanoESI MS. Species with the remark “CID” have been further characterized by CID experiments and evaluation of the resulting fragment ion spectra.
(PDF)

S1 Fig Mass spectrometric analysis. For each analysis, detected peptides are given with their aa positions (Start, End), observed monoisotopic mass of the respective peptide in the spectrum [Observed (m/z)], experimental mass of the respective peptide calculated from the observed m/z value [Mr (expt)], theoretical mass of the respective peptide based on its sequence [Mr (calc)], difference between the theoretical Mr (calc) and experimental Mr (expt) masses [delta (Da)], number of missed trypsin cleavage sites (Miss) and peptide sequences (Peptide). The dots indicate trypsin cleavage sites.
(PDF)

S2 Fig Quantitative assay of biofilm formation. Wells of representative biofilms stained with safranin.
(PDF)

S3 Fig Nucleotide and respective amino acid sequences of the *sdgA/sdgB* region (SdgA /SdgB) and the upstream of *sdgB* located IS*1181* element in strain 1061. (PDF)
