# Supplementary material for: The Plasmin-Sensitive Protein Pls in Methicillin-Resistant Staphylococcus aureus (MRSA) Is a Glycoprotein
Source: PLoS Pathog. 2017 Jan 12;13(1):e1006110. doi: 10.1371/journal.ppat.1006110 (PMC5230774; doi:10.1371/journal.ppat.1006110)
Supplement: S1 Table — Species with the remark “CID” have been further characterized by CID experiments and evaluation of the resulting fragment ion spectra. (PDF) [file ppat.1006110.s001.pdf]

S1 Table. Summary of (glyco)peptides derived from acid hydrolysis of Pls from *S. aureus* strain 1061 and detected by nanoESI MS. Species with the remark “CID” have been further characterized by CID experiments and evaluation of the resulting fragment ion spectra.

peptide	n x HexNAc	m/z_{exp}	m/z_{calc}	remark
SD	0	221.06	221.0768	
SD	1	424.15	424.1562	CID
SDSD	0	423.13	423.1358	
SDSD	1	626.20	626.2152	CID
SDSD	2	829.30	829.2946	CID
SDSDSD	0	625.19	625.1948	
SDSDSD	1	828.28	828.2742	
SDSDSD	2	1031.34	1031.3536	CID
SDSDSD	3	1234.41	1234.4330	CID
SDSDSDSD	0	825.26	825.2537	
SDSDSDSD	1	1030.36	1030.3331	
SDSDSDSD	2	1233.41	1233.4125	
SDSDSDSD	3	1436.52	1436.4919	
SDSDSDSD	4	1639.56	1639.5713	
SDSDSDSDSD	0	-	1029.3127	
SDSDSDSDSD	1	1232.38	1232.3921	
SDSDSDSDSD	2	1435.49	1435.4715	
SDSDSDSDSD	3	1638.51	1638.5509	
SDSDSDSDSD	4	1841.69	1841.6303	
SDSDSDSDSD	5	-	2044.7097	
ADSD or SDAD	0	407.12	407.1409	CID, isomers
ADSD or SDAD	1	610.21	610.2203	CID, isomers
$AD(SD)_2$	0	609.19	609.1998	
$AD(SD)_2$	1	812.27	812.2792	CID, isomers
$AD(SD)_2$	2	1015.35	1015.3586	CID, isomers
$AD(SD)_3$	0	811.26	811.2588	
$AD(SD)_3$	1	1014.33	1014.3382	
$AD(SD)_3$	2	1217.44	1217.4176	CID, isomers
$AD(SD)_3$	3	1420.49	1420.4970	CID, isomers
$AD(SD)_4$	0	1013.32	1013.3178	
$AD(SD)_4$	1	1216.41	1216.3972	
$AD(SD)_4$	2	1419.46	1419.4766	
$AD(SD)_4$	3	1622.57	1622.5560	CID, isomers
$AD(SD)_4$	4	1825.65	1825.6354	
$AD(SD)_5$	0	1215.38	1215.3768	
$AD(SD)_5$	1	1418.48	1418.4562	
$AD(SD)_5$	2	1621.52	1621.5356	
$AD(SD)_5$	3	1824.69	1824.6150	
$AD(SD)_5$	4	2027.65	2027.6944	
ADSDSDAD	0	795.25	795.2639	
ADSDSDAD	1	998.36	998.3433	
ADSDSDAD	2	1201.41	1201.4227	CID
$(AD)_2(SD)_3$	0	997.35	997.3229	
$(AD)_2(SD)_3$	1	1200.39	1200.4023	
$(AD)_2(SD)_3$	2	1403.48	1403.4817	
$(AD)_2(SD)_3$	3	1606.61	1606.5611	
$(AD)_2(SD)_4$	0	1199.41	1199.3818	
$(AD)_2(SD)_4$	1	1402.50	1402.4612	

$(AD)_2(SD)_4$	2	1605.57	1605.5406	
$(AD)_2(SD)_4$	3	1808.67	1808.6200	
$(AD)_2(SD)_4$	4	2011.81	2011.6994	
$(AD)_2(SD)_5$	0	1401.49	1401.4408	
$(AD)_2(SD)_5$	1	1604.58	1604.5202	
$(AD)_2(SD)_5$	2	1807.66	1807.5996	
$(AD)_2(SD)_5$	3	2010.80	2010.6790	
$(AD)_3(SD)_4$	0	1385.47	1385.4459	
$(AD)_3(SD)_4$	1	1588.57	1588.5253	
$(AD)_3(SD)_4$	2	1791.67	1791.6047	
$(AD)_3(SD)_4$	3	1994.77	1994.6841	
