# Supplementary material for: The Plasmin-Sensitive Protein Pls in Methicillin-Resistant Staphylococcus aureus (MRSA) Is a Glycoprotein
Source: PLoS Pathog. 2017 Jan 12;13(1):e1006110. doi: 10.1371/journal.ppat.1006110 (PMC5230774; doi:10.1371/journal.ppat.1006110)
Supplement: S1 Fig — For each analysis, detected peptides are given with their aa positions (Start, End), observed monoisotopic mass of the respective peptide in the spectrum [Observed (m/z)], experimental mass of the respective peptide calculated from the observed m/z value [Mr (expt)], theoretical mass of the respective peptide based on its sequence [Mr (calc)], difference between the theoretical Mr (calc) and experimental Mr (expt) masses [delta (Da)], number of missed trypsin cleavage sites (Miss) and peptide sequences (Peptide). The dots indicate trypsin cleavage sites. (PDF) [file ppat.1006110.s002.pdf]

Pls

Start	End	Observed (m/z)	Mr(expt)	Mr(calc)	Delta	Miss	Peptide
411	424	734,37	1466,74	1466,73	0,01	0	K.GTGNFTAHGDIIHK.T
535	546	558,79	1115,56	1115,59	0,03	0	K.TNNLSQIGAAK.V
598	619	767,71	2300,10	2300,15	-0,05	0	R.LNDVVLNYDAATSTITATYAGK.T
688	704	624,31	1869,91	1869,94	-0,02	0	R.EFDPTLEPGSPDVIVQK.G
779	795	599,64	1795,89	1795,88	0,00	0	K.NPETGEVVTPPVDDVTK.H
839	853	787,93	1573,85	1573,82	0,03	0	K.TTTTPTTINPLTGEK.V
907	923	898,96	1795,91	1795,88	0,02	0	K.NPETGEVVTPPVDDVTK.H
1073	1085	693,32	1384,63	1384,65	-0,03	0	R.VLDPTMEPGSPDK.V
1110	1141	848,46	3389,80	3389,70	0,10	1	K.VGEGEPTTEVTKEPIDEIVNYAPEIIP HGTR.E
1159	1184	683,85	2731,39	2731,36	0,03	1	K.DGLKDPETGEIIEEPQDEVIIHGAK.D

SraP

Start	End	Observed (m/z)	Mr (expt)	Mr (calc)	Delta	Miss	Peptide
560	575	912,92	1823,83	1823,84	-0,01	0	(K)MTNAGQSVTYYFTDVK(A)
576	590	757,40	1512,79	1512,81	-0,03	0	(K)APTVTVGNQTIEVGK(T)
634	650	881,44	1760,87	1760,89	-0,02	0	(K)IGQSTVTVVSTDQANNK(S)

S1 Fig. Mass spectrometric analysis. For each analysis, detected peptides are given with their aa positions (Start, End), observed monoisotopic mass of the respective peptide in the spectrum [Observed (m/z)], experimental mass of the respective peptide calculated from the observed m/z value [Mr (expt)], theoretical mass of the respective peptide based on its sequence [Mr (calc)], difference between the theoretical Mr (calc) and experimental Mr (expt) masses [delta (Da)], number of missed trypsin cleavage sites (Miss) and peptide sequences (Peptide). The dots indicate trypsin cleavage sites.
