# Supplementary material for: The Plasmin-Sensitive Protein Pls in Methicillin-Resistant Staphylococcus aureus (MRSA) Is a Glycoprotein
Source: PLoS Pathog. 2017 Jan 12;13(1):e1006110. doi: 10.1371/journal.ppat.1006110 (PMC5230774; doi:10.1371/journal.ppat.1006110)
Supplement: S2 Fig — Wells of representative biofilms stained with safranin. (PDF) [file ppat.1006110.s003.pdf]

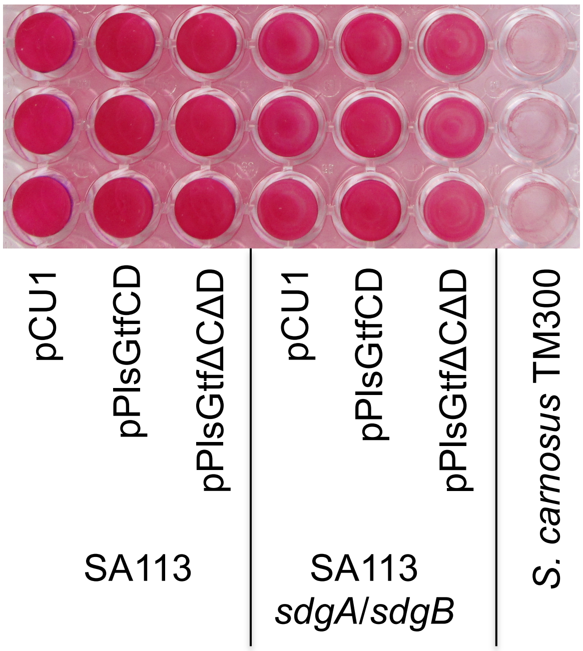

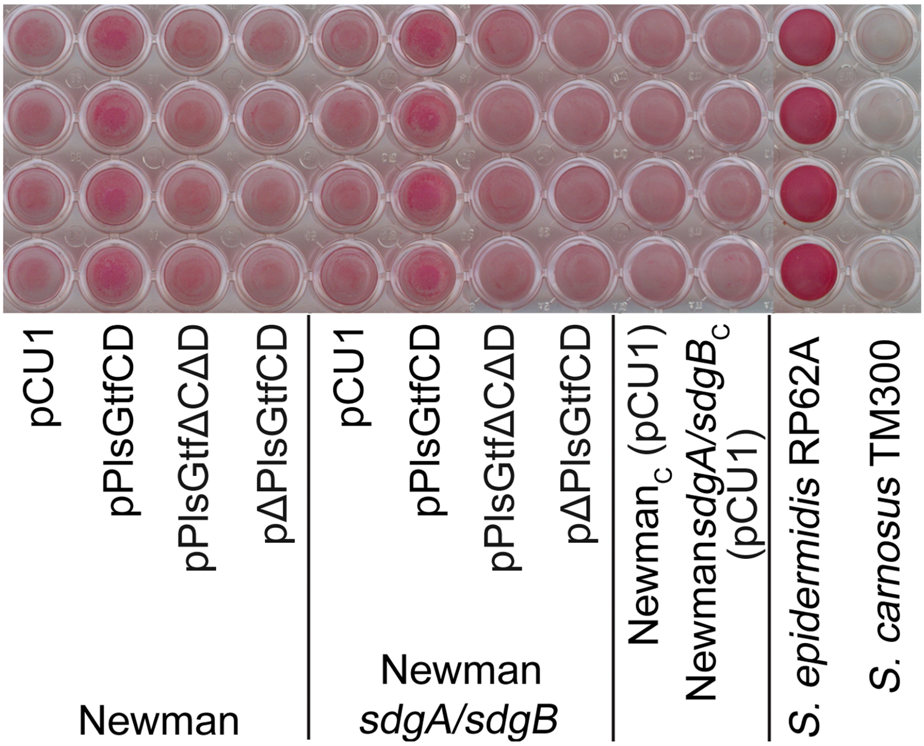

S2 Fig. Quantitative assay of biofilm formation. Wells of representative biofilms stained with safranin.
