# Supplementary material for: The Plasmin-Sensitive Protein Pls in Methicillin-Resistant Staphylococcus aureus (MRSA) Is a Glycoprotein
Source: PLoS Pathog. 2017 Jan 12;13(1):e1006110. doi: 10.1371/journal.ppat.1006110 (PMC5230774; doi:10.1371/journal.ppat.1006110)
Supplement: S3 Fig — (PDF) [file ppat.1006110.s004.pdf]

```
   1  ATATTCTAGT CGTCGCAAAA AACAAAACAA ATAATACAAT ATGACCCAGG TCCTTGTGGC CTGGTTTTTT TTATGATTAC

  81  ACATGCAATA GATGTATTTT TCATATAAAA TAAACAATAA AGATACGGAA TAAAACTTAT ATGAGGCGAT AATATGAATT
                                                                                    >>M  N

 161  ACATTTTAGG AACAATTTTA GAAAGTAAAA TTACAGGTGT AGAAAAAGCG CAAATAAATA GATTGAAGTT GTTCAAACAA
       Y  I  L   G  T  I  L   E  S  K   I  T  G   V  E  K  A   Q  I  N   R  L  K   L  F  K  Q

 241  CACGGCATAT CTTCAAAATG TGTATATGTT AAATGGAATC CTTATTCATA CACATATGCG AAGCAACATC AGATTGAAAA
        H  G  I   S  S  K   C  V  Y  V   K  W  N   P  Y  S   Y  T  Y  A   K  Q  H   Q  I  E

 321  TGATGTATTT ACAATGTATG ACTATTTTCA AAAAGCAATC AATTATAAAA AGACAAAGCA AGTTAACTGG ATACAGTATT
      N  D  V  F   T  M  Y   D  Y  F   Q  K  A  I   N  Y  K   K  T  K   Q  V  N  W   I  Q  Y

 401  GGGAAAAGTC ATGTAGGTAC ACATTGAAAT TTGTGGAAAA TTCAAATGAT GTCAGAATAT ATGATGAAGA GCAATTTGTA
       W  E  K   S  C  R  Y   T  L  K   F  V  E   N  S  N  D   V  R  I   Y  D  E   E  Q  F  V

 481  ATGTATGCTC ATTTTTTAGA TAAACAGTAT CATCAATTAA ACTATGTGAA TTATTTTGAT CATAAAAGAA GAAAAGTAAA
        M  Y  A   H  F  L   D  K  Q  Y   H  Q  L   N  Y  V   N  Y  F  D   H  K  R   R  K  V

 561  ACGCGAATTG TATGATGGAA GAGGCTTTTT AAGTTGTTCT CGAATTTTAG GCGAAGGACA ACGGATTGTA CTCGAAAATT
      K  R  E  L   Y  D  G   R  G  F   L  S  C  S   R  I  L   G  E  G   Q  R  I  V   L  E  N

 641  ACTATACACC TAATGGGGAA ATCGTCATCC AAAAATATTT CGACGATATA AAAGGAAAAA ACACGCTCAC AAAGGTTATC
       Y  Y  T   P  N  G  E   I  V  I   Q  K  Y   F  D  D  I   K  G  K   N  T  L   T  K  V  I

 721  TTAAATGAAG ACCAGCAGCA ACAATTTTTT GATACAGAAG ATGAATTAGT TCAATATTTT CTCCATCAAT TATGTAAAAA
        L  N  E   D  Q  Q   Q  Q  F  F   D  T  E   D  E  L   V  Q  Y  F   L  H  Q   L  C  K

 801  TAATGATCAA ATCATATTAG ATCGTCCTCA TGAATTAGGA AATGTTATAG CGGGATTAAA TCAAAGTATT CCAGTTGTTG
      N  N  D  Q   I  I  L   D  R  P   H  E  L  G   N  V  I   A  G  L   N  Q  S  I   P  V  V

 881  TTGTGCTCCA TAGTACACAT TTATCCGGTA CCGGTAATGG TATAAAAAGT TTTTATAAAA CAGTATTTAA TAATTTAACA
       V  V  L   H  S  T  H   L  S  G   T  G  N   G  I  K  S   F  Y  K   T  V  F   N  N  L  T

 961  CGTTATAAAG CGATTGTTGT ATCAACAGAA CAGCAATGCC AAGATATTTC ACAATATATT GAAAATAAAA TACCAGTTAT
        R  Y  K   A  I  V   V  S  T  E   Q  Q  C   Q  D  I   S  Q  Y  I   E  N  K   I  P  V

1041  CAATATTCCG GTTGGCTACG TGGCAAATTT AAAGTATCAA TTTGACATCA ATCAAAAGGA GAAAAATCAT ATCATATCAA
      I  N  I  P   V  G  Y   V  A  N   L  K  Y  Q   F  D  I   N  Q  K   E  K  N  H   I  I  S

1121  TTGCTCGCCT CGTTGAAAAT AAACAAATTA AACATCAAAT TGAAGTGATC AAGCAATTAG TAACAAAACA TCCCAATATT
       I  A  R   L  V  E  N   K  Q  I   K  H  Q   I  E  V  I   K  Q  L   V  T  K   H  P  N  I

1201  CAATTGAATA TTTATGGACA TGGAAATGGT TTGTCAGAAT ATCGACAACT TGTAGAAGAT TATCATTTAT CGGAACATGT
        Q  L  N   I  Y  G   H  G  N  G   L  S  E   Y  R  Q   L  V  E  D   Y  H  L   S  E  H

1281  TAAATTTCAT GGTTTTAAGA CGCATATTAA TGAAGAGATT GCTAAAGCAG AACTGATGTT ATCGACAAGT AAAATGGAAG
      V  K  F  H   G  F  K   T  H  I   N  E  E  I   A  K  A   E  L  M   L  S  T  S   K  M  E

1361  GTTTTGGCTT AGCAATTTTA GAGTCGCTTT CAGTAGGTAC ACCAGTGATC AGTTATGATA TAGATTATGG GCCATCAGAA
       G  F  G   L  A  I  L   E  S  L   S  V  G   T  P  V  I   S  Y  D   I  D  Y   G  P  S  E

1441  CTGATTCAAG ATGGATTTAA TGGCTATTTA GTACCTAAAG GTGACATCAA TCAAATGGTT GAAAAGGTCG ATCAATTACT
        L  I  Q   D  G  F   N  G  Y  L   V  P  K   G  D  I   N  Q  M  V   E  K  V   D  Q  L

1521  AAATAATACT CAAAAATTGC AACAGTTTTC GATTAATAGC ATAGAATCTG CACAACAGTA CAATGCAACT ACTATCAGTA
      L  N  N  T   Q  K  L   Q  Q  F   S  I  N  S   I  E  S   A  Q  Q   Y  N  A  T   T  I  S

1601  CAAAGTGGCA AAATATTTTA AACTAAGTCA AAAGAAAAAA GCATTTTCCT AGAATCATCT ATTTAACAAA CAACGTTCAT
       T  K  W   Q  N  I  L   N

1681  ACGAACTTAC ATGTTTAGTT AAATGACATA GGAAAACGCT TTTTTTTATA AAAATAAAGG TGAAGTACAT TTATATAAGA
                                                                                       I  L

1761  CGAACAAATT GGTCCCATTG TTTAATTAAC GACGCTTTAC TATATTGTTG CGCTTTTGCC AAACTACCTT TTGACAGTCG
       R  V  F   Q  D  W  Q   K  I  L   S  A  K   S  Y  Q  Q   A  K  A   L  S  G   K  S  L  R


1841  TTGTTGTACT TCAGGATGAT CAATCACATA TTTTACTTTA TCAAATAGGG CATCTTCATC ATTTTTAGTA ATTAAATAAC
        Q  Q  V   E  P  H   D  I  V  Y   K  V  K   D  F  L   A  D  E  D   N  K  T   I  L  Y

1921  CATTGAAATC AGGCGTAATC AATTCGTTAG GTCCATATTT AATATCATAA CTAATAACTG GAACACCATG TGCTAAAGAT
      G  N  F  D   P  T  I   L  E  N   P  G  Y  K   I  D  Y   S  I  V   P  V  G  H   A  L  S

2001  TCAAGTAGCG CTAAAGAGAA ACCTTCCATG TTACTTGTTA TTAAACTTAA GTAAGCATCG CTGTATTCTT GGTTTAGATT
       E  L  L   A  L  S  F   G  E  M   N  S  T   I  L  S  L   Y  A  D   S  Y  E   Q  N  L  N

2081  GCTTAAAAAG CCACGTAAGT AAACATGATT TTCCAATCCA TATTTTTGTA TCAATTCATT TAATTTTTTA CTTTCAGAAC
        S  L  F   G  R  L   Y  V  H  N   E  L  G   Y  K  Q   I  L  E  N   L  K  K   S  E  S

2161  CAAAACCATA CATATGAAGC TCTATTTTTG GGACATACGA TACTAAGCGT TTAATTAATT CAATTTGTTG ATGTAATTGT
      G  F  G  Y   M  H  L   E  I  K   P  V  Y  S   V  L  R   K  I  L   E  I  Q  Q   H  L  Q

2241  TTTTCAGGTG AATAACGAGC AACGGAAATT AATTTAACAC TGCGCTGATC TAATGTTTGG ACTGGTGTAT CAATTGTTTC
       K  E  P   S  Y  R  A   V  S  I   L  K  V   S  R  Q  D   L  T  Q   V  P  T   D  I  T  E
```

2321 ACTATAGCCG ACAGGAATAT TAACAATTGG AATAGTATGG TTAATACGTT TTTCAACATC TAATTTTTGC TGCTCAGTAG
S Y G V P I N V I P I T H N I R K E V D L K Q Q E T

2401 AAACGATAAT TGCACGATAT CGAGATAAAT TTTCAAACAT CGCTTTATAT ACATTTTTAA ATGGCGATGA ATCTAATGCA
S V I I A R Y R S L N E F M A K Y V N K F P S S D L A

2481 TCAATATTTT TAATGTGTGT ACTGTGAAGC ACAGCTACTA CTGGGATTGA CTCAGGTGTT AAGTTGAAAA TAGGTGCTGT
D I N K I H T S H L V A V V P I S E P T L N F I P A T

2561 ATACACATTA CGGTCACTGA AAAATAAATC CCCATGTTGA TATAGTTGTT TAATGAAAAA TGCGCCTAAT TCCGTTTCAT
Y V N R D S F F L D G H Q Y L Q K I F F A G L E T E

2641 TATTAAAGAA ATATTGTTTG TTAGCATAGT AAACAATAAT TTTTTGTACT TCTGGTTTGC CATCCTTGTA AGAAAAATAC
N N F F Y Q K N A Y Y V I I K Q V E P K G D K Y S F Y

2721 TTTTCTAATT TTGTGTCACC TTCTGGATTA TAGAAAAATT CACATAATGT TTGTTGTTTA TCAACAAGAA TCCTACTACA
K E L K T D G E P N Y F F E C L T Q Q K D V L I R S C

2801 ACTTAAAAAG CCACGCACAT CATAAAAATC ACGTTTTACT TTTCGTCTTT GACTATCAAA ATGATTCACA TAATCTAATA
S L F G R V D Y F D R K V K R R Q S D F H N V Y D L

2881 TACGATATTT AGGATCTTGA AAATGGGCAT ACATTAAGAA ACGCTCTTGA TCATATATTC TAAAGTCATG ACTATTTTCA
I R Y K P D Q F H A Y M L F R E Q D Y I R F D H S N E

2961 ACATGTTTTA AAGTATAATG ACATTCATCA GTCCAATACG ACAACCAGTC AAATGGTTCA TTGCGTTCTA AATATGTTGC
V H K L T Y H C E D T W Y S L W D F P E N R E L Y T A

3041 TTCTTGGAAG AAATCATACA TATTAATATA GTCAGAACTA GTAATATAAT TTTGGGCATT TCTATATAAA TATCTATTCC
E Q F F D Y M N I Y D S S T I Y N Q A N R Y L Y R N

3121 ATGACAGAAA TACACATTGC GCTGGTCTTC CCATTTCTTT AAATAAATTT AAACGATTAA TAATTGCTTT TTCTATCCCA
W S L F V C Q A P R G M E K F L N L R N I I A K E I G

3201 GTTAAATTAA CACCTAAACT ATTACCTACA AAATAATT**CA** **T**TTACAACAC CACTTATATC TATTTTTTAT AATTATATCA
T L N V G L S N G V F Y N M<<

3281 CATAATATTT AATTACTTCT TTTAACTGGA TGATGTGTTT ATTTATAAAA CAACAAATTT TGATATTTAT AATGATAGTA

3361 GTTATTCAAT CAACTACGAC CAATATATCA TGTAGAGCTT AGGATATTGA TTTATGCCTC AGGCACATCA AATGAGAAGA

3441 TTTATAAAAG AGATATACAA CTCTAGAAGG TATAATAAAA ACGCGCAACT AATGTTACGC GTTTGAATTA ATCATATGAT

3521 ATTATGGTTC TTCATCTTTT ATGGTGGGAA GGTAAAACTT CCTGCTTTTT TTAATACACA AAAGCGCAA TTGCCTCTAT

3601 AATTTAAAGT GACCAAACCC AAACTAAAGG AGACAAGTGC GCCT**ATG**TGT AATGATACCT TAGAATTACT AAGAATAAAA
>>M C N D T L E L L R I K

3681 GATGAAAATA TAAAATATAT AAACCAAGAA ATTGACGTCA TTATCAAAGG AAAAAAAGCA ACAGTGGTTA ATGCTGTACT
D E N I K Y I N Q E I D V I I K G K K A T V V N A V

3761 AACGTATAAG CCTTCGGCCT GTTATTGTTG TGGAGTTAAA AATGAAGGAC AAATTCATAA ACATGGTAAG CGTGTTTCTC
L T Y K P S A C Y C C G V K N E G Q I H K H G K R V S

3841 GTATTACTTT ACTTAAAACT CAAGGGTATA ACACATACCT CAACTTAGCT AAACAACGTT TTAAATGCTT AGAATGCAAT
R I T L L K T Q G Y N T Y L N L A K Q R F K C L E C N

3921 GGCACTTTTA CTGCTAAAAC GTCAATTGTT GATGAGTCGT GTTTTATCTC AAGATGTGTT ACTCAAAAAG TTATAGAAGA
G T F T A K T S I V D E S C F I S R C V T Q K V I E

4001 AGCTACTAAA GTTAAAACAG AGATTGATAC TGCAGAAGAT AACTGTATCT CTCCATCTAC TGTAAGTCGT ATTAGAACTA
E A T K V K T E I D T A E D N C I S P S T V S R I R T

4081 AAGCGGCTAA TTCATTACGA ATTAAACCGT TTAATTGTTT GCCAGAACAC ATCGCTATGG ATGAATTTAA AAGCGTTAAA
K A A N S L R I K P F N C L P E H I A M D E F K S V K

4161 AATGTAACTG GATCAATGAG TTTCATTTTT ATAGATAATG ATACTCATGA TGTTATAGAT ATTTTAGAAA ATAGAACTAC
N V T G S M S F I F I D N D T H D V I D I L E N R T

4241 AAGATTCTTG CGTGCCTATT TCGAGCGATT CGATTTAAAA AATCGACAAC AAGTTAAGAC GGTTACTATT GACATGTATG
T R F L R A Y F E R F D L K N R Q Q V K T V T I D M Y

4321 AACCCTATGT CCGATTATTT CGCGACCTAT TTCCTAATGC AGCTATTATT TTTGACAGAT TCCATATCGT TCAACATTTA
E P Y V R L F R D L F P N A A I I F D R F H I V Q H L

4401 AATAGGGAAC TTAATAAGTA TCGTGTACAA GTTATGAATG AATACCGTAA TAAAAAAGGA CCTAATTATA CAATTTTTAA
N R E L N K Y R V Q V M N E Y R N K K G P N Y T I F

4481 GAATAACTGG AAAGTTCTAT TGATGGATAC TAGTAAAACC ATATTTAGTA AATACAGATG GAATAAATCT TTTAAGGCTT
K N N W K V L L M D T S K T I F S K Y R W N K S F K A

4561 ATAAACGCTC ATCTGACATT GTAGAATTCA TGCTTTCAAA AGACGATATA CTACGACGCT CCTACGAACT TGTCCAAGGA
Y K R S S D I V E F M L S K D D I L R R S Y E L V Q G

4641 TTAAGAAAAG ATCTAAGGTT ATGTAATTGG CCTAAATTTA TTAATCGTTT AAATTCAGTT AGTAAAAAGT CTGTGAGTAA
L R K D L R L C N W P K F I N R L N S V S K K S V S

```
4721  AGGTGTATGG AAAGTGGTTA AATATTATAG AAAACATCAA AGGATGTTAA GAAATACAAT TTATTACCCA GCATTTAATA
      K  G  V  W   K  V  V   K  Y  Y   R  K  H  Q   R  M  L   R  N  T   I  Y  Y  P   A  F  N

4801  ATGGTGCTAT AGAAGGAATT AATAATAAGA TAAAATTAAT CAAGCGAATT TCTTTTGGTT ACAGAAATTT CAACAACTTT
        N  G  A   I  E  G  I   N  N  K   I  K  L   I  K  R  I   S  F  G   Y  R  N   F  N  N  F

4881  AAAGCACGTA TAATGATGAT TTTCAGCTTA TACAAAGGAG AAAAAAAGAA GACAACCAAG CCCAATAATG GACTGGCCGC
        K  A  R   I  M  M   I  F  S  L   Y  K  G   E  K  K   K  T  T  K   P  N  N   G  L  A

4961  CTAATAATAA AAACTCTAAA AGTTGTAATT TAAAATAGTT CTTTAAATTA TATACCCACC ACATTTGGTG GAGAACCGAT
      A

5041  ATTATTTGCG ATACTTTAAT TTAGCGAAAG CATCATGTTG ATGGATAGAC TCTTCATTAC GACATTCGAT ATCGAAACCG
```

S3 Fig. Nucleotide and respective amino acid sequences of the *sdgA*/*sdgB* region (SdgA **/**SdgB**) and the upstream of *sdgB* located** IS*1181* element **in strain 1061.**
